# Circadian rhythms in health and disease

**DOI:** 10.1186/s43556-026-00646-7

**Published:** 2026-10-23

**Authors:** Xiaoyan Liu, Xuguang Du, Shihong Qi, Lin Yuan, Liyu Liu, Kaiyu Zhang, Liqin Wang, Wanlu Zhang, Qian Liu, Min Wu, Yongye Huang

**Affiliations:** 1 Present Address: College of Life and Health Sciences, Northeastern University, Shenyang, 110169 China; 2 Present Address: Frontiers Science Center for Molecular Design Breeding (MOE), State Key Laboratory of Animal Biotech Breeding, College of Biological Sciences, China Agricultural University, Beijing, China; 3 Institute of Neurology, Jiangxi Academy of Clinical Medical Sciences, The First Affiliated Hospital, Jiangxi Medical College, Nanchang University, Nanchang, 330200 China; 4 Joint Research Center of Wenzhou Institute, University of Chinese Academy of Sciences, and the Second Affiliated Hospital, Wenzhou Medical University, Wenzhou, 325000 Zhejiang China; 5 Human Phenome Institute, Fudan University, Shanghai, 201203 China; 6 Department of Infectious Diseases and Center of Infectious Diseases and Pathogen Biology, The First Hospital of Jilin University, Changchun, China; 7 Xinjiang Key Laboratory of Reproductive Regulation and Breeding for Ruminant Livestock, Urumqi, Xinjiang, 830000 China; 8 National Key Laboratory for Swine Genetic Improvement and Germplasm Innovation, Ministry of Science and Technology of China, Jiangxi Agricultural University, Nanchang, 330045 China

**Keywords:** Clock, Neuron, Melatonin, Serotonin, Gut microbiome, Long non-coding RNA

## Abstract

Circadian rhythms, serving as a crucial temporal bridge linking neural activity and pathological biology, are regulated by central pacemaker neurons to maintain cellular homeostasis and coordinate diverse biological processes. Circadian rhythm disruption is closely associated with various diseases, including neurodegenerative disorders, metabolic disorders, and cancer. Circadian rhythms are controlled by core clock genes, such as BMAL1 and CLOCK, modulating numerous physiological processes, encompassing metabolism, immune responses, neural activity, and hormone regulation. Circadian rhythms disruptions arising from shift work, jet lag, or inconsistent sleep schedules are implicated in both the onset and progression of numerous diseases, notably Alzheimer's disease, Parkinson's disease, diabetes, and cardiovascular disease. The article highlights how circadian dysregulation alters cellular processes to potentially accelerate disease progression. Circadian disruption results in impaired immune function, further promoting disease development and resistance to treatment. Potential therapeutic applications based on circadian biology, such as chronotherapy and personalized medicine, are also discussed. Recent technological advances, embodied in wearable devices and omics approaches, are increasingly providing deeper insights into circadian biology. Further unveiling the role of circadian in health and disease opens new avenues for tailoring treatment timing to patients' biological rhythms, thereby enhancing therapeutic outcomes across various diseases. By bridging fundamental circadian mechanisms with clinical pathology, this review provides a roadmap for future chronotherapeutic interventions in precision medicine.

## Introduction

**Fig. 1:**
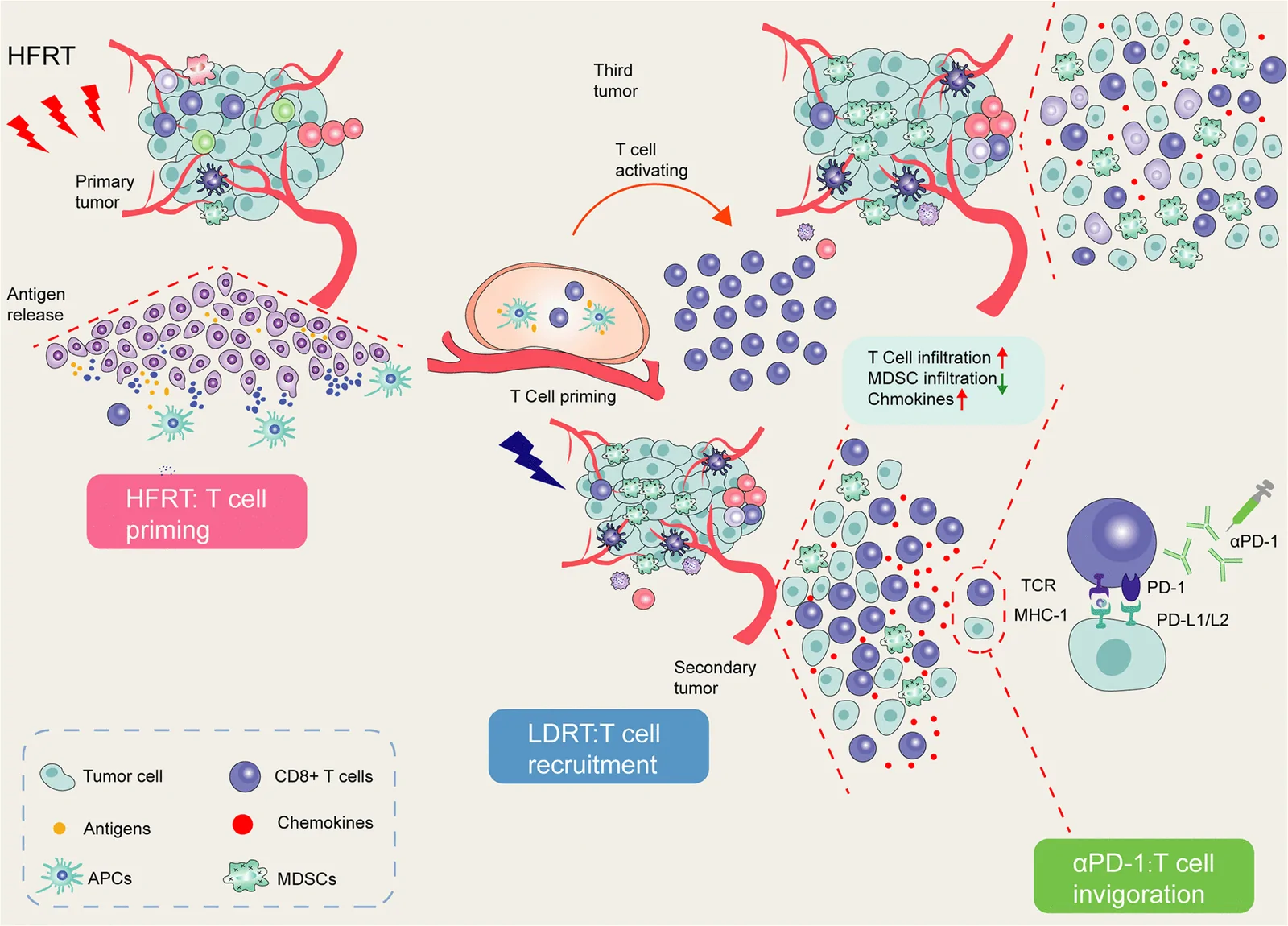
The circadian rhythm system in human physiology. Whole-body circadian organization: environmental light is perceived by intrinsically photosensitive retinal ganglion cells (ipRGCs) and transmitted via the retino-hypothalamic tract to the suprachiasmatic nucleus (SCN) master clock, which synchronizes peripheral oscillators across virtually all organ systems through hormonal signals, autonomic nervous system output (SNS and PNS), body temperature rhythms, and feeding schedules. Target organs include the heart, spleen, liver, pancreas, kidney, adrenal gland, intestine, uterus, white and brown adipose tissue, skeletal muscle, bone marrow, lymph nodes, and stem cell niches. The molecular circadian clock: the *CLOCK-BMAL1* heterodimer drives rhythmic transcription of *PER*, *CRY, REV-ERBα/β*, and *ROR* genes. *PER-CRY* complexes provide negative feedback, while *REV-ERBs* and *RORs* respectively repress and activate *BMAL1* transcription through *RORE* elements. These interlocking transcription-translation feedback loops generate approximately 24-h oscillations in clock-controlled genes (*CCGs*), *DBP*, and *NFIL3* via coordinated nuclear-cytoplasmic shuttling and proteasomal degradation of clock proteins

A robust circadian clock is crucial for maintaining cellular homeostasis, and dysregulation of circadian rhythms has been implicated in a number of complications including neurodegenerative diseases, metabolic disorders, and cancer progression [1, 2]. Mammals have evolved circadian rhythms to adapt to environmental changes in the endogenous biological clock system located in the suprachiasmatic nucleus (SCN) of the hypothalamus. This system consists of a central master clock and its location in the suprachiasmatic nucleus (SCN) of the hypothalamus and subclocks in peripheral tissues [3]. Neurons within the SCN can regulate gene expression through a transcription-translation feedback loop (TTFL) formed by core clock genes (e.g., *Clock, Bmal1, Per, and Cry*) [4]. The Clock and Bmal1 gene products form a heterodimer that promotes transcription of the *Per* and *Cry* genes, whereas the accumulation of PER and CRY proteins inhibits Clock- Bmal1 activity, thus forming a negative feedback loop ensuring a 24-h periodic oscillation. In addition, SCN neurons are synchronized by electrophysiological activity and the release of neurotransmitters (e.g., glutamate and γ-aminobutyric acid), ensuring the stability of circadian rhythms [5]. Circadian rhythms fluctuate on a 24-h cycle and regulate physiological activities, such as sleep-wakefulness, metabolism, body temperature, blood pressure, irritability, hormone synthesis and secretion [6]. Current research has illuminated that the disturbance of circadian rhythm may negatively affect the normal function and regulatory mechanisms of cells, increasing the risk of various diseases. There have also been reports revealing the mechanism of sleep deprivation leading to circadian rhythm disturbance and thus promoting tumor occurrence [7, 8]. Fatty acid oxidation (FAO) can act as a "receptor" of circadian rhythm disturbance, transforming the effect of sleep deprivation into a cancer-promoting metabolic signal in the occurrence of lung cancer [9]. Circadian rhythm disruption, caused by genetic, environmental, or pathological factors, involves desynchronization between central and peripheral clocks or altered molecular clock parameters, impairing transcriptional feedback loops, disrupting physiological rhythms, and increasing susceptibility to diseases like cancer [10]. Its operation relies on synchronized inputs from environmental signals like light to coordinate various physiological activities ranging from cellular metabolism to overall behavior (Fig. 1).

Neurons distribute across the body and could be classified into peripheral and central nervous systems. The nervous system plays a vital role in controlling the populations of tissue stem cells and precursors throughout an individual's lifespan. Neural signaling refer to the communication mechanism by which the nervous system transmits information between neurons and/or between neurons and peripheral tissues (including tumors) through electrical activity and chemical messengers (glutamate, dopamine, etc.), thereby regulating physiological functions and pathological processes. Beyond its involvement in developmental processes, the nervous system is now recognized as a crucial modulator of cancer, exerting influence from the initial formation of tumors to their malignant growth and subsequent metastatic dissemination [11, 12], forming a new field—“cancer neuroscience”. This emerging field is currently focused on exploring the role of the nervous system in cancer and potential therapeutic strategies [13]. Progress in cancer neuroscience may create an important new pillar of cancer therapy [14].

Current articles in this emerging field refer mainly to the central role of the nervous system in the pathophysiology of primary brain tumors, brain metastases, and solid tumors in systemic organs. In this review, we would like to emphasize the potential relationship among circadian rhythms, the nervous system and diseases. The dual role of circadian rhythm genes in cancer has been extensively studied, with their effects varying across cancer types and genetic backgrounds. In certain cancers, such as breast and prostate cancer, core circadian genes like *BMAL1* and *CLOCK* exhibit tumor-suppressing functions, whereas in other cancers, their dysregulation may promote tumor progression [15]. The functional consequences of circadian disruption are highly dependent on specific genetic mutations present in the tumor and environmental factors affecting the biological clock. For instance, mutations in circadian genes may increase tumor-promoting pathways, while certain genetic backgrounds may enhance the protective effects of circadian regulation [16]. Consequently, the impact of circadian rhythms on cancer biology is complex, exhibiting context-dependent effects that necessitate personalized therapeutic approaches. The possible mechanisms by which circadian rhythms directly affect the psychological recovery of cancer patients are currently focused on three aspects: common genes, synthesis and secretion of hypothalamic pituitary adrenal axis hormones, and activity in brain regions related to emotional processing. Therefore, delving into these relationships can help better understand how diseases occur, providing insight and targets for cancer prevention and treatment, representing one of several key themes explored in this comprehensive review. This article systematically examines circadian regulatory mechanisms from molecular foundations to systemic physiology, and from pathological disruption to therapeutic translation, providing an integrated perspective on how circadian biology shapes health and disease.

## Molecular and cellular basis of circadian rhythms

### Core clock genes and transcriptional–translational feedback loops

Before understanding the interaction between circadian rhythms and diseases, it is necessary to systematically elucidate their core regulatory mechanisms. These mechanisms collectively form a multi-level regulatory network. An internal timing device prescribed by organisms in the course of evolution, under the pressure of natural selection, which specifies the physiological and behavioral cycles within 24 h of a day, that is, the circadian rhythm [17]. Through the regulation of the circadian rhythm, almost all organisms on Earth are able to show periodic changes, so that organisms show better adaptability and survival ability [6]. The circadian rhythm may be related to cancer, symptom clusters, and sleep pathway effects of time patterns, emotional regulation, depression, personality traits, and other factors [18]. Indirect factors affecting the psychological recovery of cancer patients may cover a range of cancer-related symptom clusters that include a collection of symptoms such as pain, depression, fatigue, and sleep disturbances that occur during cancer treatment [19]. The symptoms of cancer patients are complex and dynamic. When sleep disorders occur, the patient manifested reduced Vagus nerve tone and decreased ability to relieve stress, hence increasing the likelihood of depression and anxiety. These negative emotions, in turn, further disrupt the patient's sleep rhythm.

At the core of this system lies a cell-autonomous molecular oscillator governed by a set of clock genes. The genetic basis of circadian rhythms lies in their core regulatory layer. Circadian regulation revolves around a central pacemaker in the hypothalamus that is synchronized with the circadian clock of peripheral tissues [20]. Each cell contains a molecular clock composed of numerous transcription factors, activators, and suppressors referred to as clock genes. These clock genes are responsible for driving the cellular oscillations that regulate balance and metabolism [21]. A significant proportion of the transcriptome expressed in a rhythmic pattern is under the control of these intracellular clocks and exhibits tissue- and cell-specific associations. A specific subset of these transcriptomes is often referred to as clock control genes [22]. The genetic basis of the biological clock can be traced to genetically important genes and regulatory pathways. Multiple genes have been shown to be involved in shaping and maintaining the homeostasis of the biological clock. These genes include period genes and clock genes, which encode proteins that build complex self-feedback regulatory loops. The majority of the core clock genes in mammals encode transcription factor proteins that play a crucial role in initiating the rhythmic expression of target genes. Some of these clock genes form heterodimer complexes, such as *CLK-BMAL1* and *PER-CRY*, which are involved in a transcriptional-translational autoregulatory feedback loop [23]. These genes play a critical role in the regulation of the biological clock cycle, and their mutation or variation may have a significant impact on the function of the biological clock. Beyond this core genetic circuitry, the circadian clock is finely tuned by a layer of epigenetic regulation, which provides the plasticity necessary for environmental adaptation and has profound implications in health and disease.

### Epigenetic and post-translational regulation of circadian machinery

Beyond chromatin modifications, non-coding RNAs (ncRNAs) constitute another critical layer of circadian rhythmicity in epigenetic regulation. They play multifaceted roles in rhythm generation, cancer progression, and neurological function. The following sections focus on three major categories of key ncRNAs, long noncoding RNAs (lncRNAs), microRNAs (miRNAs), and circular RNAs (circRNAs), and elucidate their functional mechanisms in linking the biological clock, neural activity, and tumor biology processes.

In addition to genetic regulation, epigenetic modifications provide dynamic plasticity for circadian rhythms. As previously mentioned, the core molecular mechanism of circadian rhythms namely the transcription-translation feedback system is finely regulated at the epigenetic level [24]. These mechanisms control gene expression and cellular function by adjusting chromatin structure and genome accessibility. The vast majority of the mammalian genome consists of non-coding regions, which are transcribed into various ncRNAs, including miRNAs and lncRNAs. Circadian regulators are closely linked to several genes that activate the cell cycle (e.g., Wee1, c -Myc, p20, p21, and Cyclin D1), which are regulated by influencing histone modifications and DNA methylation [25]. Furthermore, epigenetic control coordinates circadian oscillations during transcription through chromatin remodeling. The clock genes CLOCK-BMAL1 and PER-CRY complexes have been found to interact with a variety of epigenetic modifiers, namely JARID1a, EZH2, MLL1, WDR5, CBP-p300, and many histone deacetylases (e.g., NAD + -dependent enzyme SIRT1). Clock genes in Arabidopsis exhibit rhythmic transcription, a process regulated by rhythmic histone modifications [26]. The molecular mechanisms underlying circadian rhythms include a complex transcriptional/translational feedback system containing multiple regulatory circuits that are intricately regulated at the epigenetic level, which ensure the precise functioning of the biological clock and the maintenance of physiological rhythms [27].

Numerous studies have elucidated that hypermethylated down-regulation of RORA is associated with poor survival in renal clear cell carcinoma (KIRC), suggesting that hypermethylated RORA may be a cancer driver [28]. DNA methylation, particularly cytosine-guanine dinucleotides, is one of the most common epigenetic modifications. Earlier studies have detailed the effects of benzo[a]pyrene on DNA methylation, which manifests itself as either hypomethylation or hypermethylation, depending on its concentration and the response of different cells, tissues and organisms [29]. In addition, the toxicity of benzo[a]pyrene is potentially linked to the dysregulation of the biotin homeostatic pathway, which plays an important role in cancer development [30]. At the core of the methylation process, the methyl group is derived from S-adenosylmethionine and covalently attached to the 5th carbon of the cytosine ring to form 5-methylcytosine. This methylation works by modifying the promoter region of a gene, usually resulting in reduced or silenced gene expression [29]. In Arabidopsis, the circadian clock consists of three interlocking transcription-translation feedback loops. The central loop is known as the core oscillator. This loop consists of three transcription factors, TOC1, CCA1, and LHY. Early morning expression of CCA1 and LHY represses the transcription of the evening gene TOC1. Methylation of lysine residues in the tail of histone H3 is a key mechanism for regulating chromatin state and gene expression and is mediated primarily by a family of enzymes with SET structural domains that regulate the dimethylation and trimethylation of H3K4me3. H3K4me3 present in the promoter of TOC1 and functions in the context of TOC1 repression by CCA1. Previous studies have shown that JMJ proteins inhibit histone methylation and that JMJD5/JMJ30 is an important component of the Arabidopsis biological clock. In mammals, the rhythmic expression of DNA methyltransferases implies that DNA methylation plays an important role in the transcription and regulation of clock genes.

Lysine-specific demethylase 1-like 1 (LDL1) and LDL2 can also interact with CCA1 and LHY to inhibit TOC1 expression. Recent studies have shown that the FAD + -dependent histone demethylase LSD1 plays an important role in the fine regulation of histone proteins, whereas the JARID1a histone demethylase enhances CLOCK by enhancing histone acetylation. Night shift work may lead to an imbalance in the circadian rhythm of DNA repair gene expression, which can increase cancer risk. CLOCK-BMAL1 is known to recruit MLL1 to promote trimethylation of H3K4 on nucleosomes near the circadian rhythm gene promoter, thereby activating transcription [31, 32]. In contrast, EZH2 achieves transcriptional repression by methylating H3K27 at the promoter of clock control genes [33]. The histone demethylases Jarid1A and Jmjd5 also have key roles in the epigenomic function of circadian rhythms [34, 35].

Epigenetic regulation possesses a central function in regulating circadian oscillations in transcription through the mechanism of chromatin remodeling. Circadian modifications of histones, such as acetylation and deacetylation, are essential for transcription initiation and feedback repression, and these modifications serve as signaling platforms for chromatin remodeling enzymes that ensure the temporal correctness of gene expression. Post-translational modifications of chromatin regulate the compaction and accessibility of genomic elements, finely controlled by combinations of acetylation, phosphorylation, methylation, and ubiquitination, which reorganize the chromatin landscape and thus influence gene expression. Histone acetylation is the most widely studied post-translational modification in the nervous system, and histone acetylation is essential for regulating gene expression and has been implicated in a variety of neurobiological mechanisms [36]. And mammalian histone methyltransferases mediate H3K27me2 and H3K27me3 on the PER1 and PER2 promoters, which are required for mammalian rhythmic clock function. Activation of CLOCK-BMAL1 target genes in the circadian oscillator coincides with histone acetylation, showing the importance of histone acetyltransferase (HAT) activity of the transcription factor CLOCK in circadian chromatin remodeling. In addition, the CLOCK-BMAL1 complex dynamically recruits a variety of HATs including CBP and p300, which further promotes cyclic acetylation of histone tails and enhances transcription of the Per1 gene [37].

Beyond their role in orchestrating rhythmic gene expression, these ncRNAs act as critical nodes in the interface between circadian dysfunction and oncogenesis, providing potential targets for precision chronotherapy. Long non-coding RNAs (lncRNAs), defined as transcripts exceeding 200 nucleotides, are critical regulators of chromatin dynamics and gene expression within the circadian framework [38, 39]. Small RNAs, typically derived from intergenic or antisense transcripts, serve as key regulators of chromatin structure at non-circadian regulatory loci. LncRNAs function through multiple mechanisms, including recruiting epigenetic and regulatory components to target genomic sites and modulating splicing and translation [39]; when located in the nucleus, they modify chromatin structure by interacting with chromatin-modifying enzymes to alter gene expression [40]. In the circadian context, lncRNAs assist in permissive and non-permissive chromatin oscillations that require clock-mediated transcriptional support [38]. The circadian antisense lncRNA Per2AS, encoded on the antisense strand of the core clock gene PER2, regulates clock amplitude through direct interactions with BMAL1 as well as repression of PER2 transcription [41]. Deletion of lncRNA 116HG leads to dysregulation of CLOCK, CRY1, and PER2, and the high-amplitude lncRNA ADIRF-AS1, regulated by the molecular clock, binds to the PBAF chromatin-modifying complex to drive clear cell renal cell carcinoma (ccRCC) tumorigenesis [42]. A cyclic NF-κB-driven lncRNA (lc-uc) physically interacts with the CBX1 protein via H3K9me3, reducing its gene-silencing activity and enhancing transcription of Rev-erbα, thereby linking the circadian clock with colitis [43]. LncRNAs are abundantly expressed in the central nervous system and participate in neurodevelopment, brain function, and aging [44]. In Alzheimer's disease (AD), the leading cause of dementia associated with neuronal damage and cognitive deterioration, non-coding RNAs are implicated in AD pathophysiology and may play important roles in neuronal function [44, 45]. In cancer, the majority of lncRNAs are abnormally expressed in tumor cells and tissues, regulating gene expression closely linked to tumor formation, invasion, and metastasis [46, 47]. In colorectal cancer, lncRNAs synergize with microRNAs and mRNAs in biological processes such as liver metastasis, epithelial-mesenchymal transition (EMT), inflammation formation, and chemo-/radiotherapy resistance [48]. lncRNAs also show therapeutic potential in conditions such as acute lung injury [49]. Collectively, lncRNAs not only exert regulatory influence on circadian rhythms but are also controlled by the biological clock, providing new perspectives for understanding and treating various diseases.

MicroRNAs (miRNAs) are small single-stranded molecules (18–22 nucleotides) that regulate gene expression post-transcriptionally, typically by binding to the 3'UTR of target messenger RNAs [50, 51]. miRNA-target interactions exhibit high temporal and tissue specificity, participating in diverse physiological and pathophysiological processes, particularly cancer development [51]. Several miRNAs are intimately involved in circadian regulation. Molecular clock genes can modulate circadian oscillations of miR-21-5p and miR-21-3p, triggering daily apoptotic rhythms by targeting the pro-apoptotic Xaf1 and controlling molecular clock output in atherosclerotic lesions [52]. The neuron-enriched miR-183/96/182 cluster, located within a highly conserved 4-kb genomic region on chromosome 6qA3 in mice, is highly expressed in sensory organs [53, 54]. All three members regulate circadian rhythms, with miR-96 directly targeting the core clock gene PER2; knockdown of this cluster produces tissue-specific effects on circadian parameters and alters behavioral rhythms in mice [55]. In SCN neurons, miR-219–1 and miR-132, targets of the BMAL1:CLOCK complex and CREB respectively, modulate circadian rhythms by influencing PER1 transcription and PER2 protein expression [56], highlighting a direct link between miRNA-mediated regulation and neural clock mechanisms. Beyond the SCN, miR-325 expression levels are specifically associated with rhythmic sleep disorders, suggesting a key role in circadian regulation [57]. Circadian rhythms in the intervertebral disc are regulated by hsa-let-7f-1-3p through direct targeting of the 3'UTR of BMAL1 [58], while miR-25-3p fine-tunes circadian rhythmicity by modulating PER2 oscillation at the post-transcriptional level in synergy with miR-24-3p [59]. Advances in bioinformatics tools have facilitated the identification and functional characterization of these regulatory miRNAs [60]. Together, these findings establish miRNAs as critical post-transcriptional modulators linking the molecular clock to cellular physiology and disease pathogenesis [61].

Circular RNAs (circRNAs) are covalently closed non-coding RNAs generated by back-splicing that lack free 5' or 3' ends, endowing them with greater stability than linear RNA molecules [62, 63]. CircRNAs are highly expressed in the nervous system, particularly enriched in synapses [62]. Emerging evidence implicates circRNAs in circadian regulation: circRNA3503 is significantly upregulated following melatonin-induced cellular sleep [64], indicating that circRNAs may respond to neuroendocrine signals and participate in rhythmic processes. As a novel class of ncRNAs, circRNAs could serve as important regulators within the circadian system and potentially form a molecular link between neural activity and cell cycle regulation in disease progression.

MT1/MT landscape serves as a critical interface through which circadian rhythm disruption is mechanistically translated into the acquisition of hallmark cancer capabilities. This is exemplified by specific pathways where clock-associated epigenetic alterations directly drive oncogenic processes. For instance, beyond its role in KIRC, the hypermethylation and silencing of the clock-related gene RORA represents a nexus linking circadian dysfunction to immunosuppression; RORA loss can disrupt normal inflammatory signaling, thereby fostering an immune-evasive tumor microenvironment. Recently, a collaborative study demonstrated that the key circadian molecule RORA is associated with anti-tumor immunity [65]. RORA activation enhances cytotoxic T cell activity by suppressing PD-L1 transcription. The research further uncovered that the molecular mechanism by which DDX3X, a member of the DEAD-box helicase family, competes with HDAC3 for binding to RORA, thereby upregulating PD-L1 expression and exerting an immunosuppressive effect [65]. Similarly, miRNAs, like the miR-183/96/182 cluster, which are under circadian regulation and target core clock components such as PER2, can fine-tune cellular rhythms. Their dysregulation may disrupt the temporal order of cell cycle checkpoints, thereby promoting genomic instability and uncontrolled proliferation [61]. These instances underscore a fundamental principle: circadian rhythm disruption reprograms the epigenetic machinery—through DNA methyltransferases, chromatin modifiers, and ncRNAs—to enact specific transcriptional programs that confer angiogenic potential, metabolic reprogramming, tissue invasion, and immune escape. Thus, the epigenetic layer is not merely a reflection of a disrupted clock but an active executor of its oncogenic consequences. By exploring biological mechanisms of action through the genetic and epigenetic levels, scientists are laying a solid foundation for a deeper understanding of the connections between the biological clock and other areas of biology. Genetic and epigenetic regulation of the biological clock involves the interaction of multiple genes and regulatory pathways. These complex regulatory mechanisms are not only a key area of scientific research, but are also essential for understanding how organisms adapt to environmental changes and maintain internal homeostasis. Therefore, an in-depth study of these regulatory mechanisms will help us to more fully understand the function of the biological clock and its important role in maintaining the health and stability of organisms. Further investigating the interaction between genetics and epigenetics can shed light on how the biological clock affects an organism's adaptation to its environment and its role in biological diversity. This understanding will provide a scientific basis for the development of new therapeutic approaches and health intervention strategies, particularly the regulation of human physiological rhythms and the treatment of related diseases.

The molecular clock within individual cells is synchronized and its signals are disseminated throughout the organism via key neurohormones, which translate temporal information into systemic physiological responses.

### Cross-talk with metabolic and signaling pathways

Building upon the genetic and epigenetic mechanisms discussed earlier, circadian rhythms also play a pivotal role in driving cancer progression, particularly through their influence on tumor proliferation, metastasis, and the tumor microenvironment. The transformation of normal cells into cancer cells is called tumorigenesis, which is a gradual process involving the accumulation of multilevel reactions and mutations [66]. During the development of cancer, a cell's DNA may be damaged, resulting in genetic mutations. These mutations may affect the control of cell growth and division, causing cells to begin to grow and divide uncontrollably and form tumors. In addition, some carcinogens or metabolites of carcinogens have the property of inducing sudden mutations, which may induce mutations in regulatory or constitutive genes, leading to cancer. Disruption of circadian rhythms has been reported facilitating tumorigenesis and promoting the establishment of cancer characteristics [67]. A biological clock is an endogenous timekeeping system that controls and optimizes biological processes in concert with the master biological clock and peripheral clocks, and is controlled by a variety of genes [68]. In complex multicellular organisms, biological clocks coordinate several physiological processes by establishing circadian rhythms. Notably, disruption of circadian rhythms contributes to the development of many chronic diseases (e.g., cancer and metabolic disorders) [69]. Human data from the Cancer Genome Atlas show significant correlations between a set of unregulated core clock genes and oncogenic driver pathways, survival and tumor stage in 32 different cancer types [70]. It has been demonstrated that CLOCK and BMAL1 may have conserved tumor suppressor effects in humans and rodents. For instance, the loss of BMAL1 not only disrupts the cellular clock but also leads to increased intracellular ROS and alters the secretion of neuroendocrine factors, which in turn can affect the recruitment and function of immunosuppressive cells like myeloid-derived suppressor cells (MDSCs) in the TME, creating a permissive niche for tumor growth [71].

Hypoxia is a key factor driving the pathological changes of tumor microenvironment, and is a necessary condition for tumor occurrence, growth, invasion, metastasis and resistance to treatment. The study found that ROS causes many of the same changes in the tumor microenvironment as hypoxia, including tumor cell dedifferentiation and metastasis. Therefore, hypoxia/ROS-induced vascular malformations and disrupted blood flow may synergistically promote metastasis with hypoxia/ROS-induced EMT. It has previously been reported that oxygen may modulate EMT and MET in primary tumors or micrometastases, respectively, both processes involved in the development of clinically detectable metastases. ROS has been reported to cause neuronal and astrocyte death through apoptosis and necrosis mechanisms. Further studies showed that 12-o-tetradecanoylphorol-13-acetate (TPA) had a strong stimulating effect on the invasion/migration of U87 cells, which effectively inhibited the invasion/migration event of glioma cells. Prolonged hypoxia promotes the expression and activation of adenosine monophosphate activated protein kinase (AMPK) α2 subtype and the production of VEGF in glioma cell lines and glioblastoma multiforme. Thus, circadian rhythm-associated hypoxia/normoxia levels may drive dynamic but relatively long-term changes in tumor cell differentiation/ de-differentiation, which is critical for the progression of metastatic disease. *PER2 and CRY1* were found to inhibit VEGF production in tumor cells, particularly in response to hypoxia [72]. Therefore, tumor cell cycle 2 loss and circadian clock dysfunction may play an important role in pathologic hypoxia-induced high expression of VEGF, leading to accelerated tumor angiogenesis and a more aggressive tumor phenotype [73].

In addition to directly regulating the dedifferentiation of tumor cells, disruption of the pathological tumor cell clock also leads to the increase of intracellular ROS, which drives the dedifferentiation of tumor cells. We have previously described that aberrant expression of circadian clock control genes may promote glioma progression by interfering with the normal cell cycle [74]. Similarly, CRY2 deletion accelerated lymphoma development in c-Myc dependent genes [75]. However, it was previously elaborated that in the absence of p53, CRY1 and CRY2 may be absent to suppress tumorigenesis [76]. Therefore, the effects of clock defects are cancer type and clock gene-specific, and the complex interactions between clock gene mutations and circadian rhythm genes remain to be further explored.

In humans, single nucleotide polymorphisms in the CLOCK and/or BMAL1 genes have been associated with increased susceptibility to several cancer types in population-based studies such as prostate, breast, ovarian and pancreatic cancers [77, 78]. For example, some studies have shown that the circadian rhythm genes PER2 and BMAL1 play an important role in cancer [79, 80]. PER2 and BMAL1 are two important members of circadian genes involved in the regulation of the biological clock and cell cycle synchronization. The core circadian genes PER2 and BMAL1 have cell-autonomous repressive effects in tumor transformation and tumor progression. The expression of PER2 and BMAL1 is frequently altered, leading to asynchrony in cell proliferation and metastasis, and promoting tumorigenesis and progression. Many experiments have confirmed that knockdown of clock genes is closely related to tumorigenesis, such as Per mutant mice are susceptible to cancer [81]. PER2 activation leads to overexpression of the oncogene c-myc, which suppresses the expression of core clock genes and leads to increased tumor incidence [82, 83]. In addition, the nuclear receptors REV-ERBα (also known as NR1D1) and REV-ERBβ (also known as NR1D2) are nuclear receptors that constitute an important part of the circadian rhythm and are involved in the regulation of cell metabolism and cell cycle synchronization. In related cancers, the expression of Rev-erbα and Rev-erbβ is often altered, resulting in dissynchroization of cell metabolism and cell cycle, which promotes tumor occurrence and progression. Mouse models of CRY1 and CRY2 deletion showed increased incidence of lymphoma, hepatocellular carcinoma and pancreatic tumors in chronically clock-deficient animals [70, 84]. Circadian clocks regulate the expression of several secreted factors with paracrine or endocrine functions. Some of these secreted factors, including cytokines, hormones, and neurotransmitters, can influence clock function through their cognate receptors and downstream signaling pathways, thereby entraining or synchronizing the clock in different tissues. These endocrine factors can serve as biomarkers of circadian function in different tissues. However, some tumors produce excessive amounts of such biological clock-related hormones or cytokines, which can also disrupt the biological clock in distant organs [67]. Simultaneous deletion of BMAL1 and APC may lead to over-activation of WNT signaling, which potentially drives intestinal transformation, significantly altering the global gene expression program involved in driving intestinal tumor progression by affecting WNT signaling and MYC activation in order to accelerate colorectal cancer progression [1].

Through the study of the biological clock, we have found that it not only plays a core role in the regulation of the cell cycle, metabolism and immune function, but also plays an important regulatory role in the process of tumor proliferation and metastasis. Gut microbiome refers to the large community of microorganisms inhabiting the gut, which plays an important role in maintaining human health through their interactions with the host [85]. Research is increasing evidence that circadian changes in the gut microbiome can influence the development and progression of cancer. Certain gut bacteria may have a greater impact on the development and spread of cancer at certain times. Disrupted circadian rhythms can accelerate cancer metastasis. Circadian rhythm disturbance induces the accumulation of myeloid suppressor cells and dysfunctional CD8 + T cells, thus promoting cancer metastasis. In addition, dysregulated gut microbiota and its metabolite taurocholic acid also promote myeloid inhibitor cell accumulation, leading to cancer metastasis [86]. Gut microbes can also influence nervous system function and cancer development. There are interactions between the gut microbiome and the brain that affect brain function through neural, immune and endocrine pathways [87]. Some gut microbes can influence the synthesis and metabolism of neurotransmitters, which can affect aspects of mood, cognition, and behavior. Therefore, the progress of circadian regulation of gut microbes in cancer neurology is a research area full of challenges and opportunities.

A large number of microbial community habitats in the intestinal tract of animals, which help the host to perform a variety of physiological and biochemical functions. These microorganisms occupy different ecological niches in the gut, competing and cooperating with each other to maintain a relative balance. The immune system plays a key role in this balance. Some bacteria are considered probiotics in the gut and they have a positive impact on the immune system. Probiotics, such as Lactobacillus and Bifidobacterium, can help regulate the activity of the immune system and promote normal immune cell function. They enhance the host's resistance to pathogenic microorganisms by competitively excluding pathogenic bacteria and modulating the activity of immune cells [88, 89]. Administration of Lactobacillus plantarum IS-10506 has been shown to increase interleukin (IL)−4 and IL-17 levels in adults with atopic dermatitis [90]. Furthermore, Lactobacillus plantarum IS-10506 modulates the immune system by decreasing serum levels of IL-17, IL-4 and interferon (IFN)-γ in children with atopic dermatitis [91].

The intestinal immune system is an important component of the human immune system and there is a close interaction between it and the intestinal flora. The intestinal immune system consists of lymphoid tissue, immune cells and mucosal barriers beneath the intestinal mucosa. These components work together to ensure that pathogenic microorganisms cannot easily cross the intestinal mucosa and enter the body. At the same time, the intestinal immune system needs to recognize and tolerate normal microorganisms in the intestinal flora so as not to over-activate the immune response. As the largest and most complex micro-ecosystem in the human body, the gut microbes themselves and their metabolites not only regulate human health, but also play an important role in bridging the gap between the diet and the host. As Nobel Prize winner Joshua Lederberg once pointed out, the human body and its symbiotic microbes constitute a superorganism. Meanwhile, the animals follow a circadian cycle, and the feeding cycle allows the animals to be rhythmically exposed to food-borne pathogens. Collectively, the innate immunity of the gut of animals may exhibit a circadian rhythm to resist pathogens that invade the host with food.

The relationship between the gut microbiota and sleep has been studied since the last century. Gut microbial metabolites influence host central and hepatic clock gene expression and sleep timing, and regulate body composition through circadian rhythm transcription factors. Activation of the hypothalamic–pituitary–adrenal (HPA) axis has now been shown to influence the composition of the gut microbiota [92]. Specifically, stress and HPA axis activation were associated with a decrease in Lactobacillus and Anaplasma spp. and an increase in Clostridium spp [93]. Also, chronic stress can disrupt the integrity of the intestinal barrier [92]. However, signaling between the gut microbiota and the HPA axis is bidirectional. Exposure to stress early in life alters brain-gut axis function [94]. Therefore, a healthy circadian rhythm is helpful to maintain the balance of intestinal microbiota and improve the function of the immune system for the prognosis of a variety of diseases.

In summary, metastasis is not an autonomous process but one meticulously orchestrated by the host nervous system and circadian rhythm system. From EMT to implantation, each step is permeated by the regulation of neural signals (such as neurotransmitters and electrical activity) and rhythmic signals (such as clock genes and hormone fluctuations). Understanding this complex spatiotemporal regulatory network not only reveals the biological basis of metastasis but also uncovers previously unrecognized targets for developing time-based therapies or neuromodulatory treatments that block specific metastatic steps.

### Neurohormone

Neurohormones serve as key messengers linking the nervous system to the biological clock. Neurohormones hold an important function in regulating circadian rhythms [95], and two key neurohormones are melatonin and serotonin. Endogenous rhythms of melatonin secretion are produced by the suprachiasmatic nuclei and are aligned with the light/dark cycle. Melatonin is capable of transmitting information about the daily light/dark cycle to body structures, which is used to organize various physiological functions and enable the body to respond accordingly to the photoperiod, thereby promoting sleep by regulating the circadian rhythm of certain physiological functions in the body [96, 97]. In mammals, the neurohormone melatonin, after being secreted during the night, acts primarily on melatonin MT1 and MT2, two G protein-coupled receptors [98, 99]. It has been hypothesized that melatonin promotes sleep by activating MT1 receptors in the SCN through the hypothalamus, a key brain region in the neural circuitry that regulates sleep and produces arousal [99]. Melatonin secretion is not only affected by exogenous factors like light and season, but may also be influenced by endogenous factors including gender, age, and puberty. Since the mid-1980s, humans have been using melatonin supplements to treat circadian rhythm sleep disorders by altering the timing of circadian rhythms, and studies have found that melatonin reduces sleep latency in the blind, ameliorates delayed sleep stage syndrome, and regulates the sleep–wake cycle [100]. In a study involving seven totally blind subjects with free-running circadian rhythms who took 10 mg of melatonin one hour before their preferred bedtime each day for three to nine weeks, the average circadian cycle during the melatonin trial was significantly different from that during the placebo trial, suggesting that melatonin was effective in controlling circadian rhythms in these subjects [101]. It is widely believed that a single dose of melatonin in the early evening can advance circadian rhythms in humans. At the same time there are studies that confirm that melatonin administration causes phase shifts and/or stabilization of circadian rhythms [102].

The circadian system is highly correlated with neurotransmitters such as serotonin, and studies have confirmed that serotonin (5-hydroxytryptamine). Recently, Richard Naud's group at the University of Ottawa in Canada reported on the expected code of value in the serotonin system, an important step in our understanding of the role of serotonin in learning and behavior [103]. Serotonin is a regulatory neurotransmitter, and therefore serotonin is likely to be a key factor in the dysregulation of circadian rhythms [104]. For example, developmental deletion of Pet-1, a transcription factor that determines the phenotype of 5-hydroxytryptamine neurons, disrupts the rhythms of locomotor activity and prolongs the period and amplitude of SCN neuronal activity in vivo [105]. Circadian regulation of 5-hydroxytryptamine requires input from the sympathetic nervous system. It has been well documented that stress-induced changes in glucocorticoids may affect the 5-hydroxytryptamine system, thereby interfering with normal circadian function [106, 107]. The importance of 5-hydroxytryptamine in the regulation of sleep has been demonstrated in numerous experiments over the last century. In experiments with cats, for example, inhibition of 5-hydroxytryptamine synthesis by parachlorophenylalanine or destruction of 5-hydroxytryptamine-containing neurons in the dorsal midbrain resulted in insomnia for more than 48 h [108]. Collectively, neurohormones have a crucial function in circadian rhythm regulation, allowing adaptation to different day and night environments and maintain a healthy body clock and sleep–wake cycle by acting directly on the central nervous system, regulating the endocrine system and the retina.

5-hydroxytryptamine is a neurotransmitter that is an important signaling molecule in the periphery, especially in the gastrointestinal tract. 5-hydroxytryptamine affects the nervous system and immune system in an autocrine or paracrine manner [109, 110]. 5-hydroxytryptamine is involved in regulating a wide range of physiological and psychological activities in the body, including sleep, mood, and memory. Serotonin is associated with the regulation of circadian rhythms. Recent studies have shown that serotonin is likely to influence the development of cancer [111]. The role of serotonin in the regulation of circadian rhythms may lie in influencing cancer development by regulating the processes of proliferation and apoptosis of tumor cells [112]. Several studies have found that serotonin synthesis and release are regulated by circadian rhythms that may be associated with the growth and apoptotic processes of tumor cells. For example, some tumor cells are more active at night, and serotonin synthesis and release also typically increase at night. Thus, by regulating the synthesis and release of serotonin, the growth and apoptotic processes of tumor cells can be influenced. Ferroptosis known to be a newly discovered form of programmed cell death, distinct from apoptosis and necrosis, associated with cancer immunotherapy and tumor suppression. Liu et al. demonstrated that the tryptophan metabolites 5-hydroxytryptamine and 3-HA significantly enhanced the anti-iron death activity of tumor cells at relatively low concentrations [113]. What is known is that 5-hydroxytryptamine receptors are expressed in a variety of cancer types, including colorectal cancer, pancreatic cancer, hepatocellular carcinoma, breast cancer and adenocarcinoma of lung [114, 115], and promote the growth and survival of tumor cells. High systemic 5-hydroxytryptamine levels are found in multiple tumors, which is closely related to tumor recurrence and poor survival. 5-hydroxytryptamine stimulation in normal and colorectal cancer patients showed that 5-hydroxytryptamine could promote the proliferation of colorectal cancer cells, but did not increase the mitosis rate of normal colon crypt cells [116]. All these evidences suggest that 5-hydroxytryptamine is closely related to tumor development.

5-hydroxytryptamine is also a potent angiogenic factor in tumor angiogenesis. Low concentrations of 5-hydroxytryptamine can induce endothelial cell proliferation, migration and tube formation in vitro, and angiogenesis in vivo is dependent on TR3/Nur77, whereas 5-hydroxytryptamine receptors are not dependent on vascular endothelial growth factor A (VEGF-A) [117]. Most of the body's 5-hydroxytryptamine is stored in platelets. In the TME, platelet aggregation leads to a massive release of 5-hydroxytryptamine and promotes tumor microvessel formation. Furthermore, the presence of 5-hydroxytryptamine-positive cells was associated with higher microvessel density and VEGF expression [118]. During tumorigenesis, the release of vascular growth factor in the TME promotes the increase of vascular growth and vascular remodeling. Compared with wild-type mice, the microvascular density of colon cancer allografts from TPH-deficient mice was reduced. The expression of VEGF and VEGFR2 was similar, but the expression of matrix metalloproteinase 12 (MMP-12) and angiostatin was higher in TPH-deficient mice [119]. MMP-12 may cleave plasminogen to angiostatin, an endogenous inhibitor of angiogenesis [120]. One study reported that 5-hydroxytryptamine acts as a vasoconstrictor that selectively constricts small tumor arteries, thereby inhibiting the growth of colon cancer.

Serotonin may influence cancer development by modulating the tumor immune microenvironment. Several studies have shown that 5-hydroxytryptamine can affect the activity and function of immune cells, including macrophages and lymphocytes [121]. These immune cells play an important role in the tumor immune microenvironment and can influence the growth and apoptotic process of tumor cells. Therefore, by regulating the synthesis and release of serotonin, the tumor immune microenvironment can be influenced, which in turn affects the development of cancer. Reduced growth and increased accumulation of functional CD8 + T cells in pancreatic and colorectal cancers in peripheral blood 5-hydroxytryptamine-deficient (Tph1-/-) mice subcutaneously and in situ injected with homozygous mice, resulting in prolonged overall survival, compared with control mice [122]. New research has found that 5-hydroxytryptamine synthesis levels can both alter and be altered by gut flora [123]. Enterochromocytes contain more than 90% of the body's 5-hydroxytryptamine, and the synthesis and release of 5-hydroxytryptamine from enterochromocytes are regulated by short chain fatty acids produced by spore-forming Clostridium difficile [124]. The stimulatory effect of these microorganisms on enterochromaffin cells increased with increasing utilization of dietary tryptophan. Analysis of plasma metabolite profiles in germ-free mice showed that 5-hydroxytryptophan levels were more than two-fold lower in germ-free mice compared to conventionally colonized mice, and that SCFAs and 2BAs from intestinal spore-forming bacteria had a significant modulatory effect on the synthesis and release of ECC 5-hydroxytryptamine. The essential amino acid tryptophan is a key molecule in the brain-gut axis because it is a precursor of the neurotransmitter 5-hydroxytryptamine and many other metabolites that are involved in neuroendocrine signaling within the brain-gut axis [125]. Based on this, the close connection between gut microbes and tumor neuroimmunity we will continue to discuss below.

Other circadian hormones, such as testosterone and glucocorticoids, also determine the dynamics of tumor cell production [126]. Glucocorticoids are steroid hormones produced by the end products of the hypothalamic–pituitary–adrenal (HPA) axis, and the production of glucocorticoids has a circadian rhythm [127]. The hypothalamus produces glucocorticoids in response to stimulation of neurons in the paraventricular nucleus. The HPA axis is known to be activated during circadian patterns and stress responses, and pro-inflammatory cytokines and pathogen activation of pattern recognition receptors also trigger glucocorticoid synthesis [128]. When they are released into the circulation, glucocorticoids inhibit further activation of the HPA axis. acute activation of the HPA axis is required to suppress immune hyperactivation. Prolonged glucocorticoid signaling is particularly relevant to cancer, where chronic pre-tumor infections, cancer-related psychological stress, corticosteroid therapy, or intra-tumor steroidogenesis suppress anti-tumor immune responses and promote tumor cell survival [129]. Adrenocorticotropic hormone, which promotes the production and release of cortisol and corticosterone, was also higher in the plasma of metastatic mice. Elevated levels of cortisol and corticosterone were associated with increased tumor volume [130]. Glucocorticoids may inhibit the recruitment of additional leukocytes to sites of inflammation by blocking the production of chemokines, histamine, and prostaglandins and by reducing the expression of adhesion molecules such as integrins and selectins [131]. Glucocorticoids also inhibit the expression of pro-inflammatory cytokines such as IL1B, TNF, IFNG and IL6, while promoting the expression of anti-inflammatory cytokines such as IL10 and TGFB. Dysregulation of these inflammatory processes is associated with cancer development [132]. For example, approximately 90% of gastric cancers are associated with long-term H. pylori infection. Chronic inflammation is a necessary driver of H. pylori-associated gastric cancer. Immunodeficient mice are resistant to H. pylori-associated gastric disease [133]. Disruption of glucocorticoid signaling has been reported to be associated with an increased risk of gastric cancer [134]. Given their anti-inflammatory effects, glucocorticoids may inhibit gastric precancerous inflammation. In a carbamate lung tumor model, glucocorticoid treatment inhibited lung tumor development, whereas adrenalectomy significantly increased the number of tumors [135]. Cigarettes are the leading risk factor for lung cancer, and glucocorticoid treatment has been shown to reduce the risk of cancer in mice exposed to cigarette smoke [136]. Therefore, glucocorticoid therapy that reduces inflammation in the lungs may prevent the development of cancer. Glucocorticoids also induce the expression of TSC22D3 (GILZ), thereby reducing the expression of pro-inflammatory cytokines and inhibiting the presentation of antigens to CD8 + T cells, thus inhibiting the anti-tumor effect of dendritic cells [137]. Within the lymphocyte compartment, glucocorticoids directly inhibit CD8 + T cell activation by driving the expression of the immune checkpoint inhibitors TIGIT, PD1, TIM3, and LAG3 and by promoting the expression of the anti-inflammatory cytokine IL10 [33]. CD8 + T cells are key agents of anti-tumor immunity, and inhibition of these cells by inducing checkpoint proteins can significantly promote tumor growth and metastasis. Immunostimulatory therapies, such as ICBs, aim to suppress these checkpoint proteins and promote anti-tumor immunity. For example, melatonin inhibits c-MYC and CYCLIN D1 expression in tumor cells via MT1/MT2 receptors, thereby blocking cell cycle progression [138, 139]. Simultaneously, it activates the SIRT1/PGC-1α pathway to improve mitochondrial function and enhance oxidative stress defense—mechanisms particularly pronounced during nocturnal melatonin secretion peaks [140]. Dopamine, via the DRD2 receptor, inhibits VEGFR2 phosphorylation and downstream ERK/FAK signaling, thereby suppressing angiogenesis and tumor invasion [141]. Its circadian secretion oscillations may amplify this inhibitory effect during active periods (such as daytime in humans), consequently influencing the spatiotemporal growth patterns and therapeutic response of tumors. Altogether, the influence of neurohormones regulating circadian rhythms on tumorigenesis is a complex process involving a variety of neurohormones and correlates of tumorigenesis.

## Circadian regulation in physiological homeostasis

### Neural activity and sleep–wake regulation

Beyond its cellular timekeeping role, the circadian system acts as a central integrator that orchestrates the daily rhythms of major physiological processes, including the sleep–wake cycle, metabolism, and immune function, thereby maintaining systemic homeostasis. This coordination is achieved through both neural projections and humoral signals. Notably, the nocturnal surge of melatonin, synthesized from serotonin in the pineal gland in response to diminishing light, directly promotes sleep onset and consolidates sleep architecture by acting on MT1 and MT2 receptors in the SCN and other brain regions [142, 143]. Concurrently, the circadian rhythm of core body temperature, peaking during the active phase and reaching its nadir during sleep, facilitates the initiation and maintenance of sleep.

### Hormonal rhythms and endocrine control

Melatonin, which is produced in the pineal gland by serotonin via the tryptophan-serotonin-melatonin biosynthetic pathway, is a neurohormone that maintains circadian rhythms by synchronizing with environmental cues, and is involved in a wide variety of physiological processes, including blood pressure and core body temperature regulation, oncogenesis, and immune function [144, 145]. The pineal gland is sensitive to light and begins to secrete melatonin when the light decreases and stops when the light is strong [146]. Serotonin is a key neurotransmitter in the central nervous system that regulates emotional stability, anxiety, and depression through a variety of receptors, such as 5-HT1A, 5-HT2A, and others. 5-HT1 receptor ‌could couple with Gi/o protein, it inhibits adenylate cyclase activity, reduces intracellular cAMP levels, and participates in processes such as mood regulation and inhibition of neurotransmitter release. For example, activation of the 5-HT1A receptor can inhibit the release of norepinephrine and participate in mood regulation [147].

### Immune surveillance and inflammatory responses

Recent studies have illuminated that the clock component REV-ERBα has a strong anti-inflammatory function in addition to its role in circadian rhythms [148, 149]. Macrophages and monocytes exhibit strong circadian rhythms, which are implicated in the control of metabolic functions and cytokine production in these cells. Similarly, rhythmic effector function affects the severity of autoimmune diseases: mice lacking BMAL1 in T cells show decreased severity scores after induction of experimental autoimmune encephalomyelitis [150], and mice with CLOCK loss-of-function mutations lose rhythmic expression of IL-23R in γδ T cells, exhibit decreased IL-22 expression, and have significantly reduced psoriasis severity [151]. Several cytokines and intercellular signaling molecules (e.g., IL-6, IL-12, TNF-α) exhibit circadian rhythms, with their concentrations varying at different time points during the day. Interleukin-1 (IL-1), one of the earliest cytokines produced in the acute phase reaction of infectious diseases, also exhibits sleep-regulating properties: in healthy individuals, IL-1 increases non-rapid eye movement (NREM) sleep characterized by increased EEG delta wave power, and similar somnogenic effects are observed during acute infections such as influenza. The circadian regulation of these cytokines affects immune cell activity, the intensity of the immune response, and the regulation of the inflammatory response. Collectively, these findings underscore that transcription factors and clock components play a crucial role in autoimmune and inflammatory diseases.

**Fig. 2:**
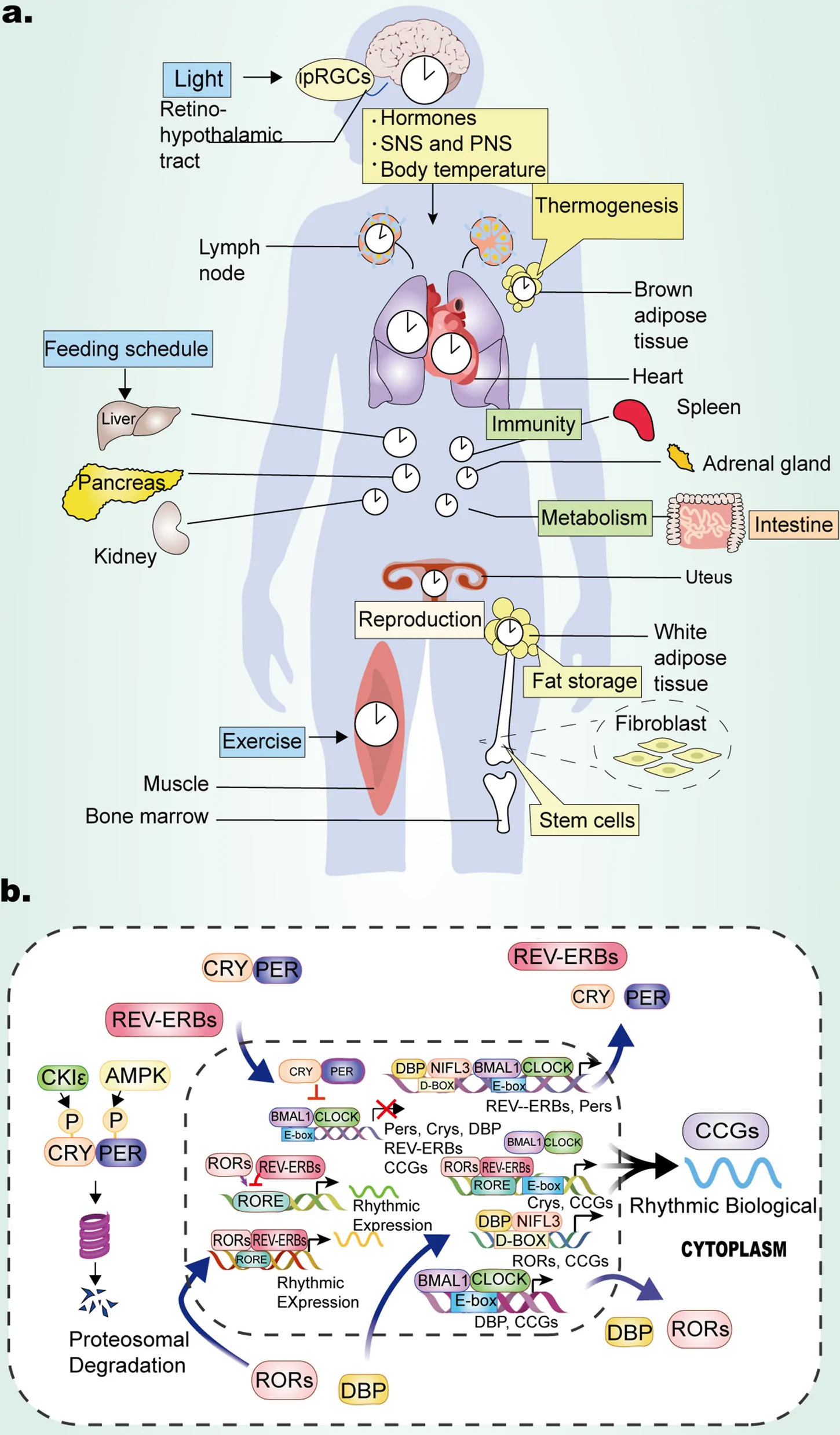
The tumor microenvironment (TME). **a** The *TME* is the internal environment for the generation and survival of tumor cells, comprising cellular components (tumor cells with abnormal proliferation and metabolic capabilities, cancer-associated fibroblasts secreting growth factors, and immune cells with dual pro- and anti-tumor roles) and non-cellular components (extracellular matrix providing physical support and biomolecules involved in intercellular signaling). **b** Through the interplay between hypoxia and immunosuppression, chronic inflammation, intercellular signaling (cytokines, growth factors, exosomes), and epigenetic regulation (DNA methylation, histone modification), these components collectively influence tumor growth, invasion, metastasis, and immune escape. The *TME* represents a complex ecosystem, and understanding its mechanisms provides new insights and targets for tumor therapy

Building on these principles of circadian immune regulation, the intersection of circadian immunity and the tumor microenvironment (TME) represent a critical nexus where clock-controlled immune functions directly influence cancer progression. The TME is the cellular environment in which tumors exist and is a key factor in tumorigenesis, development, metastasis, and treatment, consisting of tumor cells, immune cells, stromal cells, and vascular cells [152, 153]. The circadian clock exerts important influences on the formation and function of the tumor microenvironment (Fig. 2). Circadian pattern subtypes refer to different patterns of clock gene expression in the body, which show different expression levels at different times of the day; these subtypes can affect the body's immune system function and disease risk. Some findings have shown that circadian disruption alters the pattern of the immune microenvironment, thereby promoting tumor growth [154]. Chronic circadian dysregulation has been reported to affect the endogenous clock of macrophages, as well as immune phenotype and function [155, 156]. Studies have revealed that chronic sleep deprivation may lead to immunodeficiency, and prolonged exposure to nighttime light in night-shift workers leads to decreased melatonin levels and disruption of clock genes, resulting in methylation of several genes including CRY2 and CLOCK [157, 158]. Circadian rhythm disruption can promote tumor growth by modulating immune responses and cell cycle regulators such as the CLOCK-BMAL1 complex, a key molecular switch in glioblastoma that controls the association between tumor stem cells and endothelial cells. For example, glioblastoma cytokines recruit macrophages to form a TME that promotes tumor progression, and the CLOCK-BMAL1 complex has been shown to be critical for maintaining GSC stemness and inducing microglial cell infiltration of GBM TME [159]. One hallmark of cancer is unresolved inflammation recruiting immune cells to the tumor [160]. Consistently, there is growing evidence that most immune cells promote tumor progression when present in the TME, and their interaction with other components can influence tumor cell progression [161]. In addition to macrophages, tumor-associated neutrophil/granulocyte MDSCs facilitate tumor progression by promoting angiogenesis, metastasis, and attenuating anti-tumor immunity [162]; tumor-infiltrating immune cells are also an important component of TME [163]. Research also suggests that the biological clock influences the efficacy of immunotherapy by modulating the function and interaction of immune and stromal cells in the TME. For instance, circadian rhythm pattern subtypes have been found to influence the efficacy of immune checkpoint inhibitors such as CTLA-4 and PD-1 in tumor therapy [164, 165]. In addition, the biological clock influences the function of tumor cells and immune cells by regulating the cell cycle, metabolism, and signaling pathways, thereby affecting the efficacy of immunotherapy [166]. At the cellular level, mitochondrial metabolism regulates DC antigen processing, and the molecular clock of DC mitochondrial metabolism governs antigen processing function and subsequent T cell activation [164]. It has been reported that TME enhances immunosuppression of macrophages by secreting cytokines such as IL-4, and factors released by macrophages (e.g., epidermal growth factor) augment cancer cell movement and invasion [167, 168]. Furthermore, the subpopulations that make up the immune microenvironment undergo dynamic ontogenetic and functional changes during tumor development, leading to tumorigenic and immunosuppressive microenvironments [169]. Signaling by GSCs not only affects the intrinsic biology of the cell (e.g., stemness) but also regulates the TME through circadian control, including transcriptional activators (e.g., CLOCK-BMAL1 complex) and repressors (e.g., CRY1 and CRY2, PER1, PER2 and PER3, and Rev-erbα/NR1D1). GSCs can promote tumor angiogenesis through different mechanisms, such as secretion of pro-angiogenic factors and extracellular vesicles, and differentiation to pericytes. Gene deletion of the CLOCK-BMAL1 complex in GSCs impairs tumor angiogenesis, and angiogenesis-associated genes (PF4, ITGAV, LPL, and POSTN) are regulated by the CLOCK-BMAL1 complex [159]. This multifaceted role of the CLOCK-BMAL1 axis in GBM exemplifies how a core circadian node orchestrates tumor progression across multiple biological tiers. At the molecular and cellular level, this complex maintains GSC stemness and drives pro-angiogenic transcription. At the TME level, it fosters an immunosuppressive niche by modulating immune cell recruitment and function [170]. Ultimately, at the phenotypic and therapeutic level, the integrity of this circadian circuitry (including downstream effectors like CRY1) determines tumor growth kinetics and response to chemotherapeutic agents such as temozolomide. Thus, BMAL1 acts not merely as a cellular timekeeper but as a central signaling hub that translates intrinsic circadian signals into systemic outputs governing angiogenesis, immune evasion, and therapy resistance, thereby fueling malignant progression in an integrated manner. For instance, recent studies in glioblastoma have revealed that the absence of BMAL1 disrupts the cell's autonomous circadian rhythm and leads to elevated levels of reactive oxygen species (ROS) within the cells as well as abnormal secretion of pro-inflammatory factors such as IL-6. These abnormal signals activate the hypothalamic–pituitary–adrenal axis, causing disordered rhythmic secretion of glucocorticoids. The elevated glucocorticoids then drive the recruitment and functional activation of myeloid-derived suppressor cells (MDSCs) in the TME, inhibiting the function of CD8 + T cells by expressing signaling molecules such as ARG1 and iNOS, thereby establishing a strong immunosuppressive barrier and promoting tumor growth [71].

**Fig. 3:**
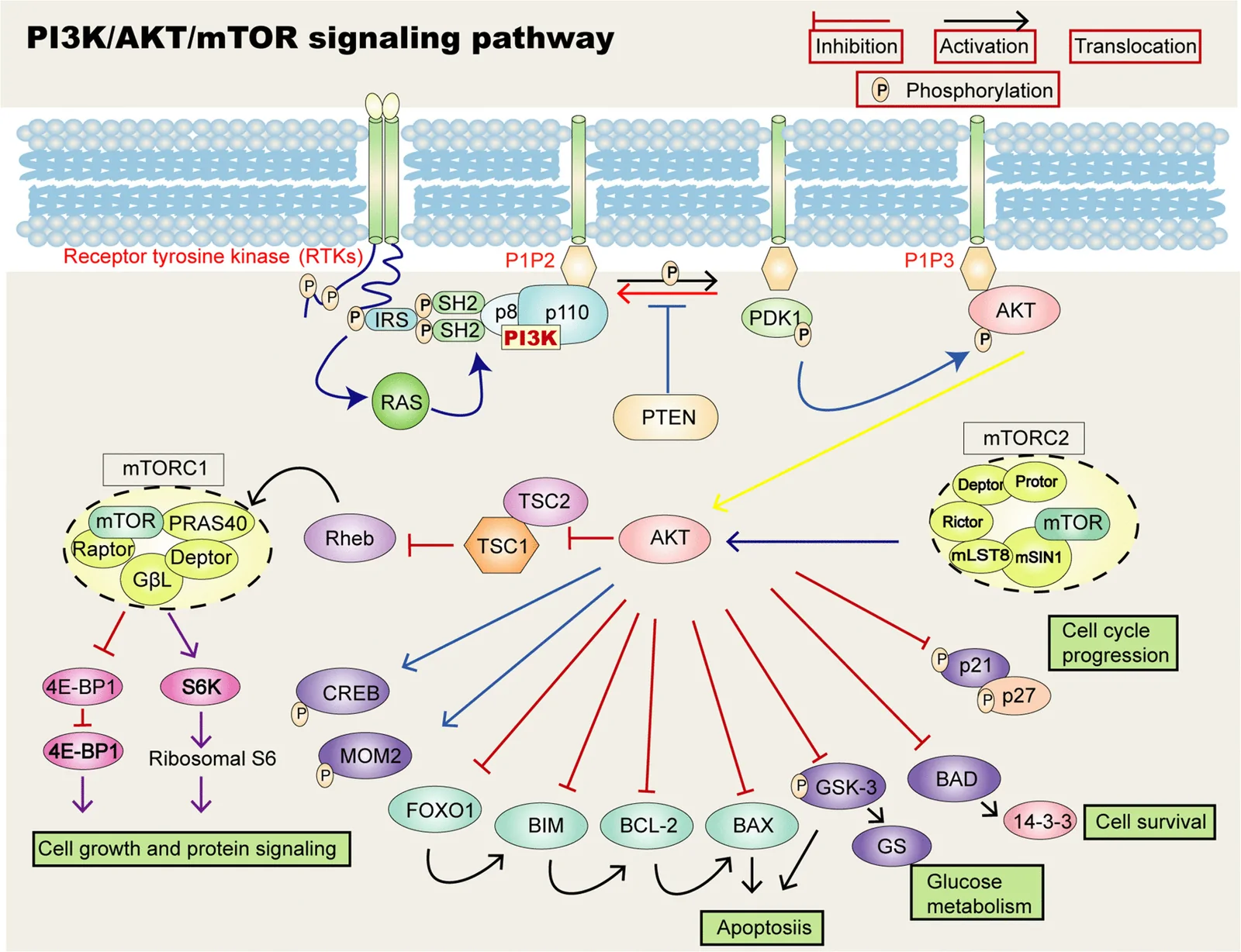
Interaction between tumors and the immune system. The immune system recognizes and attacks tumor cells via cytotoxic T cells and natural killer cells to control tumor development through immune surveillance. Tumor cells evade immune surveillance through multiple mechanisms, such as expressing *PD-L1* to bind *T* cells and releasing immunosuppressive factors. This dynamic process encompasses immune recognition and attack, tumor immune escape, and the balance between these opposing forces that determines disease outcome. Understanding this equilibrium is critical for developing effective immunotherapies that shift the balance toward anti-tumor immunity

Therefore, circadian rhythms play a crucial role in maintaining normal immune function and immune surveillance in the TME. The immune system recognizes and eliminates tumor cells, while tumor cells achieve immune evasion through various mechanisms (such as expressing immune checkpoint molecules like PD-L1), constituting the core of tumor immune editing (Fig. 3). As will be discussed in detail in Sect. 5, the interplay between circadian disruption and immune dysfunction within the TME creates a permissive environment for cancer progression and represents a promising target for chrono-immunotherapeutic strategies.

### Metabolism and energy balance

Metabolic homeostasis is under stringent circadian control at both systemic and cellular levels. Peripheral clocks in major metabolic organs including the liver, pancreas, adipose tissue, and skeletal muscle, exhibit robust oscillations that govern the timing of glucose production, insulin sensitivity, lipid metabolism, and energy expenditure. The hepatic clock, for instance, drives the rhythmic expression of genes involved in glycolysis, gluconeogenesis, and bile acid synthesis, ensuring that nutrient processing is synchronized with anticipated feeding-fasting cycles [4, 5]. Similarly, the circadian regulation of insulin secretion by pancreatic β-cells and insulin sensitivity in peripheral tissues creates a temporal window of optimal glucose tolerance, typically during the active phase. This temporal compartmentalization of anabolic and catabolic processes prevents metabolic conflict; for example, fatty acid oxidation and autophagy are often upregulated during the fasting (resting) phase, while nutrient storage and utilization are prioritized during the feeding (active) phase. The master clock in the SCN aligns these peripheral metabolic rhythms through autonomic nervous system output and hormonal signals, such as glucocorticoids. This hierarchical coordination ensures efficient energy harvest, storage, and utilization, thereby maintaining metabolic equilibrium. When external cues (such as mistimed food intake) or internal clock dysfunction disrupt this temporal order, it predisposes the organism to metabolic disorders, such as obesity, diabetes, and non-alcoholic fatty liver disease [171].

The immune system, a critical defense mechanism, exhibits profound circadian oscillations in its cellular composition and functional state. Most of the body's physiological functions, including those of the immune system, are regulated by the biological clock. The immune system is an important defense line against pathogens, and its defense effect varies with time. The three major defenses of the human immune system, non-specific immunity, humoral immunity and cellular immunity, are all impacted by the biological clock. The immune system exhibits periodic changes in function and activity at different time points. Researches have shown that this system is controlled by circadian rhythms [172]. Circadian rhythms have important implications for the regulation of the immune system and are closely related to the sleep–wake cycle and other biological rhythms [173]. The number and activity of immune cells differed at night from daytime, the number of circulating leukocytes in mammalian blood exhibits oscillations, and the number of immune cells including lymphocytes, natural killer cells and macrophages also increases relatively at night. For instance, it has been experimentally validated that the number of leukocytes in nocturnally active white rats and humans peaks during resting phases [150]. Multiple studies in the last decade have found that circadian rhythms are associated with the onset and progression of autoimmune diseases [19]. Furthermore, the effector function of leukocytes and the proliferation of activated immune cells are both time-dependent [172, 174]. This circadian rhythm regulates the number and function of immune cells and may be an adaptive mechanism for the body in fighting infection and inflammation.

This precise temporal regulation of immunity represents an evolutionary adaptation that optimizes defense against pathogens while minimizing collateral inflammatory damage during vulnerable periods, such as sleep. Recent research in chrono-immunology has further elucidated how intrinsic clocks within immune cells govern their differentiation, trafficking, and effector responses. Disruption of these immune rhythms is increasingly linked to the pathogenesis of autoimmune diseases and altered the susceptibility to infections.

At the organismal level, the most conspicuous output of the circadian clock is the regulation of sleep–wake behavior. The central pacemaker of this system, SCN is located in the hypothalamus and coordinates the 24-h rhythm of the body's physiological processes. The SCN synchronizes peripheral clocks in various tissues to ensure proper timing of critical functions like sleep onset, body temperature regulation, and hormone secretion [175]. This synchronization not only facilitates restorative sleep but also supports essential cognitive processes, including synaptic homeostasis, metabolic waste clearance, and memory consolidation. This tightly coupled system ensures that the restorative functions of sleep, including synaptic homeostasis, metabolic clearance, and memory consolidation, occur during the optimal biological time window [176].

### Organ-specific oscillators and inter-organ communication

Circadian rhythms regulate a variety of physiology and behavior, including sleep–wake cycles, hormone levels, daily changes in body temperature, blood pressure, food intake, and impairments in cellular metabolism [177]. In mammals, the SCN, a small region located in the anterior part of the hypothalamus, is the central regulator of circadian rhythms throughout the brain and body, controlling the circadian rhythms of the entire organism by regulating the rhythms of individual tissues and cells in the body [178]. The synchronization of clock neurons within the SCN occurs through the release of glutamate, triggered by light, from retinal projections that reach the hypothalamus via the retino-hypothalamic tract. Molecular clock networks are essential for daily physiology and maintenance of body health. In the latest study, researchers created a basic clock module involving a central clock and a peripheral (muscle) clock in mice with arrhythmia that showed premature aging. The interaction and importance of intra-tissue clock network in the maintenance of muscle tissue were revealed [179].

## Circadian disruption and human disease

Circadian disruption arises from diverse etiologies ranging from genetic mutations in core clock genes to modern lifestyle factors such as shift work and chronic jet lag. These disruptions propagate through multiple hierarchical levels of biological organization, from molecular clock dysfunction within individual cells to systemic desynchrony between central and peripheral oscillators, ultimately manifesting as increased susceptibility to a broad spectrum of diseases. This section provides a systematic overview of how circadian disruption contributes to neurodegenerative disorders, metabolic and cardiovascular diseases, and immune dysregulation, highlighting both shared and disease-specific mechanistic pathways.

### Genetic mutations, shift work, and social jet lag as sources of disruption

Disruptions to the circadian system, such as those caused by shift work, jet lag, or irregular sleep schedules, have profound consequences on both cognitive and metabolic health. Experimental evidence indicates that circadian disruption impairs spatial exploration and memory functions, while altering metabolic pathways such as tryptophan‑5‑HT metabolism, which is closely linked to neurotransmitter signaling and cognition [180]. These disturbances impair the ability to maintain consistent sleep quality and timing, which are essential for optimal cognitive performance. Sleep serves as a critical period for memory consolidation, allowing for the stabilization of new memories and the integration of learned information. Metabolic processes, such as the clearance of toxins from the brain, are also optimized during sleep. When this timing is misaligned, as is often seen in individuals exposed to irregular sleep patterns, it can lead to cognitive deficits, including reduced attention span, impaired decision-making, and slower reaction times. Moreover, chronic circadian disruption has been linked to metabolic disorders such as obesity, diabetes, and cardiovascular diseases.

The synchronization of circadian rhythms with the sleep–wake cycle has also been found to influence cognitive flexibility and the ability to process complex information. For example, individuals who experience circadian misalignment may struggle to adapt to changing tasks or environments, as their cognitive resources are depleted due to the insufficient restorative effects of sleep [181]. As a result, maintaining a consistent sleep–wake schedule is not only essential for physical health but also for maintaining cognitive function and memory performance, especially in high-demand situations such as academic testing or decision-making in complex environments.

### Sleep disorders and neurodegenerative diseases

Sleep disorders, including insomnia, sleep apnea, and circadian rhythm disturbances, have been increasingly recognized as significant contributors to the development and progression of neurodegenerative diseases. Disrupted sleep patterns are commonly observed in patients with Alzheimer’s disease (AD), Parkinson’s disease (PD), and other forms of dementia. In AD, poor sleep has been shown to exacerbate cognitive decline and accelerate amyloid plaque deposition in the brain, a hallmark of the disease. Furthermore, sleep deprivation can impair the clearance of neurotoxic proteins, such as beta-amyloid, by disrupting the brain's waste removal system, known as the glymphatic system. This has raised the hypothesis that sleep disorders could directly contribute to the pathophysiology of neurodegenerative diseases, possibly by impairing the brain's ability to clear toxic waste products during sleep, thereby accelerating neuronal damage [182].

In addition to its role in cognitive function, sleep is crucial for maintaining neural plasticity and brain health. In Parkinson’s disease, disrupted sleep patterns are frequently seen, particularly REM sleep behavior disorders (RBD), which are often a precursor to PD. The loss of normal REM sleep is thought to contribute to the progression of motor symptoms in PD patients by exacerbating neuronal loss in areas of the brain that control motor function, such as the substantia nigra [183]. Moreover, studies have shown that treating sleep disturbances in PD patients can improve both motor and non-motor symptoms, suggesting that sleep therapy could be an effective adjunct treatment for neurodegenerative conditions [184]. Recent evidence also indicates that sleep disorders may influence the onset and progression of other neurodegenerative diseases, including Huntington’s disease and amyotrophic lateral sclerosis (ALS) [185]. In these conditions, altered sleep regulation, such as fragmented sleep and changes in sleep architecture, have been found to correlate with worsening symptoms and faster disease progression. Research suggests that restoring normal sleep patterns could potentially slow down the neurodegenerative process, highlighting the importance of sleep in maintaining overall brain health and function.

### Metabolic and cardiovascular diseases

Circadian rhythms play a fundamental role in regulating the body’s metabolic processes, including glucose metabolism, lipid synthesis, and energy expenditure. Disruption of these rhythms, such as through irregular sleep patterns, shift work, or jet lag, has been shown to contribute significantly to metabolic disorders like obesity, type 2 diabetes, and metabolic syndrome. The liver, as a central organ in metabolism, exhibits its own circadian rhythm, regulating key processes such as glucose production and fat storage. Misalignment of the body’s biological clock, such as in night-shift workers, can lead to insulin resistance and impaired glucose tolerance, both of which are hallmarks of metabolic diseases. Additionally, research has demonstrated that circadian misalignment results in disturbed lipid metabolism, which contributes to the development of atherosclerosis and other cardiovascular diseases (CVD) [186].

The cardiovascular system itself exhibits intrinsic circadian rhythms that govern critical aspects such as blood pressure, heart rate, and vascular tone. The SCN (suprachiasmatic nucleus) serves as the central pacemaker, synchronizing peripheral clocks in organs like the heart, liver, and blood vessels. These rhythms are essential for optimal cardiovascular health, ensuring that blood pressure and heart rate are appropriately regulated according to the body’s needs throughout the day. Disruptions in circadian rhythms, such as those seen in individuals with irregular sleep–wake cycles, have been linked to increased risk of hypertension, arterial stiffness, and heart disease. Studies indicate that circadian disruptions, particularly those induced by shift work, can lead to elevated sympathetic nervous system activity, resulting in higher blood pressure and increased oxidative stress. This, in turn, leads to vascular inflammation, endothelial dysfunction, and an increased risk of atherosclerosis [187].

Moreover, circadian disruption is also implicated in the development of systemic inflammation, a key factor in the progression of both metabolic and cardiovascular diseases. Research has shown that circadian rhythm disturbances increase inflammatory markers such as C-reactive protein (CRP), which are known to contribute to atherosclerosis and other cardiovascular conditions [188]. Furthermore, the disruption of circadian rhythms can impair the function of immune cells, including macrophages and T cells, leading to chronic low-grade inflammation, which exacerbates metabolic diseases like obesity and type 2 diabetes.

### Immune dysregulation and inflammatory disorders

The immune system, a crucial line of defense against pathogens and internal threats, is profoundly influenced by the biological clock. Clock genes and their associated transcription factors are present in nearly every cell of the innate and adaptive immune system, including macrophages, T cells, and B cells. Clock genes and their associated transcription factors not only control the timing of immune cell activation but also regulate the intensity and duration of inflammatory processes. Clock genes and associated transcription factors are present in almost all cells of the innate and adaptive immune system [189]. Defects in clock genes are associated with alterations in various inflammatory phenotypes, suggesting that circadian rhythms constantly have a critical role in the normal functioning of the mammalian immune system. For example, circadian disruption can lead to an overactive immune system, characterized by excessive cytokine production and uncontrolled immune cell activation, which can contribute to autoimmune disorders and chronic inflammatory diseases [190]. Experiments have demonstrated that BMAL1 also regulates macrophage morphology and motility, influencing their ability to respond to inflammatory signals [191]. Deletion of BMAL1 results in rhythmic dysregulation of clock genes [27]. Relevant experiments have identified BMAL1 as a potent regulator of macrophage morphology and motility [150]. These genes regulate immune responses by controlling the timing and intensity of inflammatory processes, such as cytokine production and immune cell trafficking. Disruptions in circadian rhythms, particularly through the dysregulation of core clock genes, can lead to altered immune functions and an increased susceptibility to inflammation-related diseases.

## Circadian regulation in cancer neuroscience

**Fig. 4:**
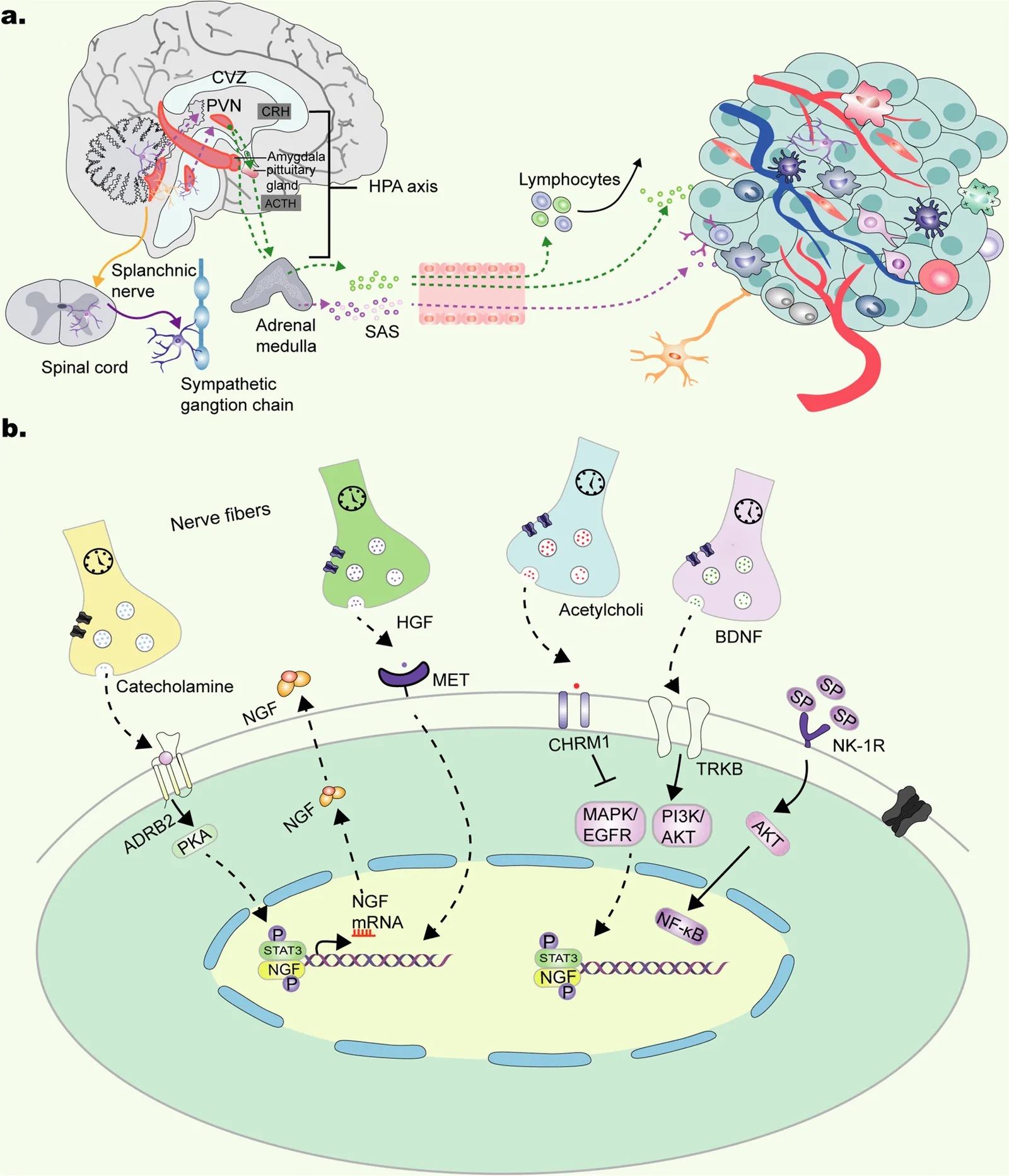
Cancer neuroscience: neuro-endocrine-immune crosstalk in the tumor microenvironment. **a** The central neuroendocrine axis: the circumventricular zone (*CVZ*) and paraventricular nucleus (*PVN*) of the hypothalamus coordinate stress responses via CRH-releasing neurons projecting to the median eminence, activating the *HPA* axis and sympathetic-adrenal-medullary (*SAM*) axis through the splanchnic nerve and sympathetic ganglion chain, while lymphocytes traffic between peripheral lymphoid organs and the TME. **b** Neuron-to-cancer cell signaling at the molecular level: nerve fibers release neurotransmitters and growth factors that bind cognate receptors on cancer cells, including catecholamines (*ADRB2/PKA* pathway), NGF (*TrkA*), HGF (*MET*), acetylcholine (*CHRM1*), BDNF (*TRKB*), and substance P (*NK-1R*), activating downstream *MAPK/EGFR, PI3K/AKT, NF-κB*, and *STAT3* pathways to drive tumor proliferation, invasion, and metastasis. These interactions span multiple levels, from direct electrochemical communication between neurons and tumor cells to the indirect regulation of the systemic tumor microenvironment by the neuroendocrine system

Cancer neuroscience is a rising area of the interactions between cancer and the nervous system, encompassing neurons, glial cells, neurotransmitters, and the interrelationships of the nervous system with cancer, including direct paracrine and electrochemical communication between neurons and cancer cells, as well as indirect interactions through neural actions on the immune system and stromal cells in the TME in a variety of malignancies [192, 193]. With the emergence of "cancer neuroscience", more and more research has begun to delve into the complex interactions between various cancers and the nervous system [194, 195]. From neural invasion of cancer cells to neurological symptoms caused by the tumor itself or its treatment, these phenomena not only reveal the intricate crosstalk between cancer and the nervous system, but are also strongly associated with poor patient outcomes and a significant increase in the burden of diagnosis and treatment [196]. There may be potential interactions between multiple metastatic cancers and central nervous system neurons, e.g., excitatory neurotransmission may play a facilitating role in brain metastases, and neurotransmitter receptor expression is upregulated in breast cancer brain metastatic cells [197]. It is well known that the TME plays a crucial role in malignant tumors, and neurons have become a key component of the TME, promoting the tumorigenesis of various cancers [198, 199]. Neuronal activity is emerging as a driver of cancer in the central and peripheral nervous system, and growth of central, and peripheral nervous system tumors has been reported in Nf1 mutant mice [200]. Also, nerves may regulate the proliferation, migration, and invasive capacity of cancer cells through the release of various neurotransmitters or factors [201]. Various clinical medical models have demonstrated that the activity of the nervous system can control the development of cancer and exert a strong influence on its progression and metastasis [202, 203]. At the same time, cancer can also reshape the harmony and hold the structure and function of the nervous system [204]. The disruption of circadian rhythms leads to the disruption of biological clock function, and the generated tumor promoting metabolism may increase the likelihood of tumor occurrence and development. Recent studies have enriched our understanding of the role of the nervous system in carcinogenesis, which regulates tumor growth and cancer-related symptoms through neuroendocrine pathways. For example, the hypothalamic–pituitary–adrenal (HPA) axis and hypothalamic-pituitary-growth hormone (GH) axis lead to abnormal release of hormones [205, 206]. These hormones activate signaling pathways that upregulate the expression of downstream oncogenes, which in turn affect the immune system and the regulation of tumor growth [202, 206]. Neurons determine physiological changes related to cancer through the involvement of the HPA axis, through the production of glucocorticoids (which in turn act on the immune system), and through the innervation of the autonomic nuclei of the brainstem, which activate the sympathetic nervous system, leading to increased activation of the cerebral cortex and alterations in the circadian rhythms; these signals also appear to be involved in cancer pathogenesis [207]. Compared to normal tissue, the circadian rhythm of tumor cells seems to be somewhat disrupted. The clock genes are widely mis-expressed in various human cancers. Importantly, the degree of error in clock gene expression seems to be closely related to the staging or invasion of various cancers. If the biological clock is disrupted in the late stage of Hodgkin's lymphoma. Compared with all other breast cancer, the three groups of negative breast cancer had more clock gene misexpression [208]. Moreover, the clock and cell differentiation are closely intertwined, and the core clock is not clearly manifested in undifferentiated pluripotent stem cells, but gradually manifests as cell differentiation and biological development progress. Therefore, it can be hypothesized that some circadian clock regulatory factors can act as tumor suppressors. In addition, neurotransmitters and neurohormones also play an important role in nervous system-cancer interactions, and recent evidence suggests that neurotransmitters such as epinephrine, norepinephrine, and glucocorticoids, dopamine, and 5-hydroxytryptophan promote tumorigenesis and progression [137, 206]. Glucocorticoids promote ionizing radiation-induced tumorigenesis by attenuating tumor suppressor P53 protein levels and function; prolactin promotes breast cancer invasion by activating the PRL-SRC-FAK-MAPK pathway; dopamine activates the DR1-cAMP-PKA pathway and enhances tumor vascular stability, thereby increasing the efficiency of cisplatin delivery to tumors and inhibiting tumor growth in ovarian cancer. Overall, the results of cancer neurology research can provide a basis for clinical diagnosis, treatment and prognosis. By studying the interactions between the nervous system and cancer, more effective anticancer drugs as well as neurological modulation methods for cancer-related symptoms may be developed. Therefore, cancer neuroscience, as an emerging interdisciplinary field, centers on systematically deciphering the intricate bidirectional interaction network between the nervous system and cancer. These interactions span multiple levels, ranging from direct electrochemical communication between neurons and tumor cells to the indirect regulation of the systemic tumor microenvironment by the neuroendocrine system (Fig. 4). These representative mechanisms are summarized in Table 1.

**Table 1:** Representative mechanisms linking circadian clock gene dysregulation to neuroendocrine alterations and immunosuppression in the tumor microenvironment

Category	Circadian factor / node	Representative molecular/cellular mechanism	Neuroendocrine consequence	Immune effect within TME	Tumor-related outcome	References
Core clock genes	BMAL1 (ARNTL) downregulation	Impaired CLOCK–BMAL1 transcriptional activity; dampened rhythmic control of cell-cycle and stress-response programs	Dysregulated HPA-axis output; altered glucocorticoid rhythmicity	Reduced cytotoxic immune tone; enrichment of immunosuppressive populations	Immune evasion and accelerated tumor growth; reduced treatment responsiveness	[209]
Core clock genes	PER2/PER1 downregulation	Loss of circadian constraint on proliferation and DNA damage response; disruption of clock-controlled checkpoints	Reduced circadian neurohormone rhythmicity; heightened stress-related autonomic outputs	Increased immunosuppressive signaling; impaired effector immunity	Enhanced angiogenesis/invasion potential and metastatic permissiveness	[210]
Core clock genes	CRY1/CRY2 dysregulation	Altered checkpoint control and transcriptional timing; effects may depend on tumor genetics	Stress hormone signals under circadian rhythm disorders	Suppressed immune surveillance and impaired cytotoxic activity	Facilitated tumor initiation/progression under specific genetic backgrounds	[211]
Core clock genes	REV-ERBα (NR1D1) disruption	Weakened repression of inflammatory/metabolic programs; disturbed rhythmic control of cytokine-related transcription	Blunted endocrine rhythmicity influencing systemic inflammatory tone	Skewed inflammatory milieu and immune dysfunction; potential immune exhaustion signatures	Tumor-promoting inflammatory microenvironment and progression	[212]
Core clock genes	CLOCK hyperactivity or mis-expression	Aberrant activation of clock-controlled gene programs, including proliferation/metabolic outputs	Increased autonomic/catecholaminergic drive	Bias toward tumor-supportive myeloid phenotypes	Increased angiogenesis, invasion, and immune escape	[213]
Neurohormonal signal	Melatonin deficiency / dampened rhythm	Loss of circadian anti-oxidative and anti-proliferative signaling; reduced MT1/MT2 pathway tone	Decreased nocturnal melatonin peak; impaired circadian entrainment	Reduced NK and CD8⁺ T-cell functional capacity; weaker anti-tumor surveillance	Increased tumor growth and metastatic permissiveness	[214]
Stress axis	Cortisol rhythm disruption	Chronic or mistimed glucocorticoid exposure altering immune transcriptional programs	Persistent HPA-axis activation; elevated stress signaling	T-cell dysfunction/exhaustion and broad immunosuppression	Tumor progression and poorer prognosis in susceptible contexts	[178, 215]
Autonomic signaling	Sympathetic hyperactivation	β-adrenergic pathway activation	Increased norepinephrine signaling	Recruitment of MDSCs; reduced antigen presentation	EMT, invasion, and metastatic dissemination	[178]

### Neuronal network between circadian rhythms and glioma initiation

Neurodevelopmental mechanisms and the nervous system play a crucial role in the occurrence and development of brain tumors. Here, we focused on glioma, a common intracranial malignant tumor and the most aggressive type of brain tumor, characterized by rapid proliferation and aggressive infiltration [216, 217]. Circadian rhythm is a time-regulating mechanism in organisms that controls many physiological and behavioral processes. The relationship between brain tumor occurrence and circadian rhythms has not been fully defined. It has been shown that the mammalian molecular clock is not only expressed in pacemaker neurons of the SCN of the primary visual field. In the central SCN of the hypothalamus, neuronal circadian oscillators act as the organism's master pacemaker and also drive the rhythm of biophysiological activity [218]. However, growing evidence points to that circadian rhythms have an impact on the pathophysiology of gliomas [219, 220]. We early described gliomas integrating synaptically into neural circuits. The interaction between neurons and gliomas is bidirectional; The activity of neurons increases the growth of gliomas, and gliomas are thought to increase the activity of neurons [221]. Studies have shown that human neural structure promotes the growth of glioma cells through the paracrine growth of neurogliin-3 neuronal protein [4, 159]. Similarly, non-synaptic autocrine and paracrine actions of neurotransmitters have been implicated in the progression of gliomas and other tumor entities [222]. In particular, glutamate binds to different kinds of glutamate receptors to enhance the proliferation, survival, and aggressiveness of tumor cells [223]. Glutamate signaling activates AMPA and NMDA receptors on tumor cells, forming the basis for neuron-glioblastoma network communication and directly influencing tumor proliferation and invasiveness.

**Fig. 5:**
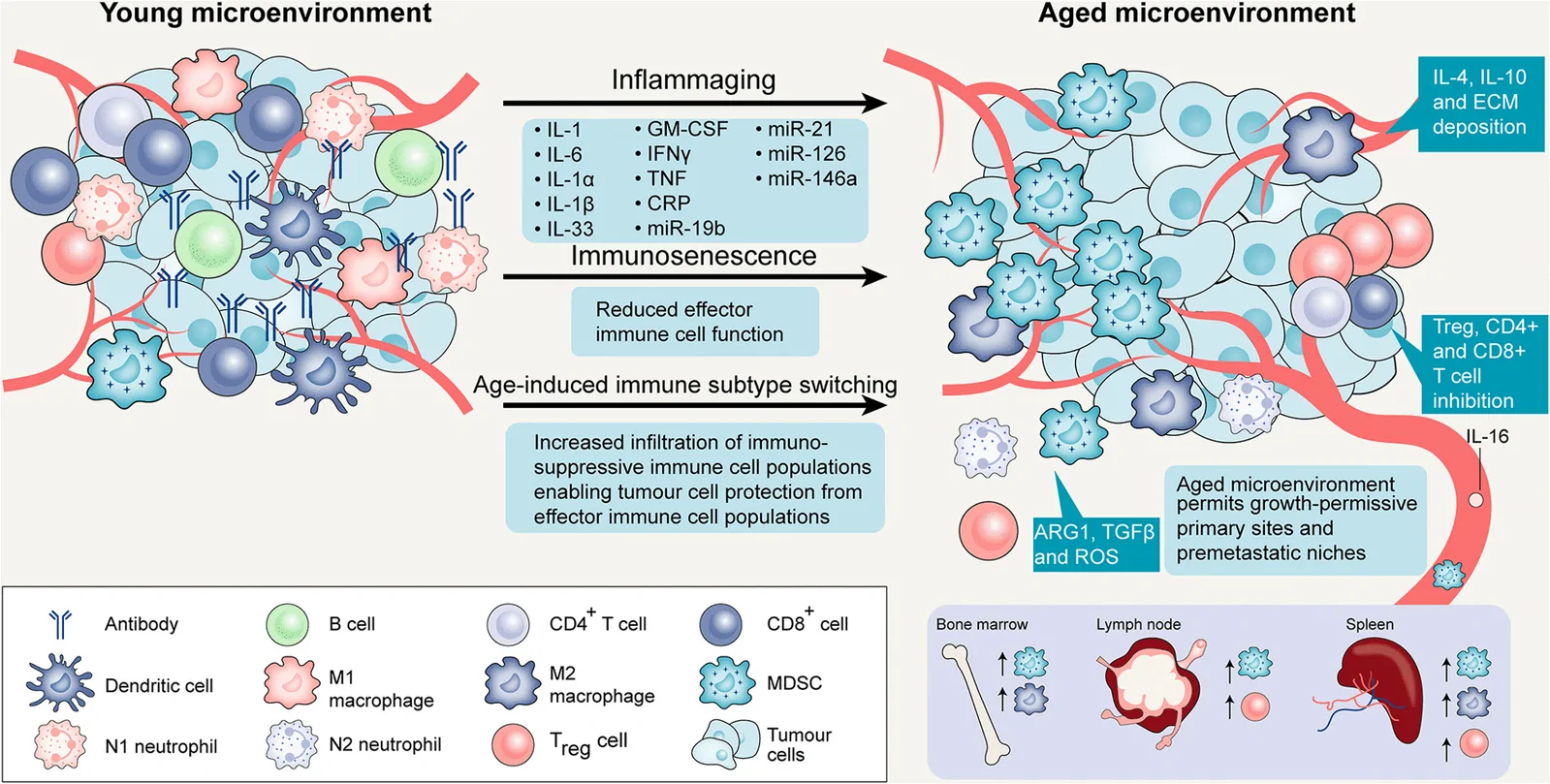
PI3K/AKT/mTOR signaling pathway. Activation: receptor tyrosine kinases (*RTKs*) recruit *PI3K* (p110/p85 heterodimer), which phosphorylates *PIP2* to generate *PIP3* at the inner plasma membrane. *PDK1* and *mTORC2* phosphorylate and activate *AKT*, while the tumor suppressor *PTEN* negatively regulates the pathway by dephosphorylating *PIP3*. Downstream signaling: activated *AKT* phosphorylates numerous substrates to control cell survival (*BAD, BCL-2, BIM, BAX* via *FOXO1* inhibition), glucose metabolism (*GSK3/14–3-3*), cell cycle progression (p21, p27, MDM2, CREB), and protein synthesis. *AKT* relieves *TSC1/TSC2*-mediated inhibition of Rheb, allowing *mTORC1* (*Raptor, PRAS40, Deptor, GβL*) activation and downstream phosphorylation of ribosomal *S6* and *4E-BP1* to drive cap-dependent translation. *mTORC2* (*Rictor, Protor, Deptor, mLST8, mSIN1*) provides positive feedback by phosphorylating *AKT* at Ser473. Aberrant activation of this pathway drives tumorigenesis, metabolic reprogramming, and therapy resistance, representing an important target for drug development

Glutathione is an important antioxidant with multiple functions, mainly maintaining cellular redox homeostasis. Glutathione retains adequate levels of cysteine and detoxifies exogenous sources while also conferring therapeutic resistance to cancer cells [224]. Elevated levels of glutathione in tumor cells can protect these cells in bone marrow, breast, colon, larynx, lung and glioma cancers by conferring resistance to several chemotherapeutic agents [225]. Carcinogenic PI3K signaling has recently been shown to stimulate glutathione production in breast cancer cells [225]. Elevated glutathione has been experimentally shown to promote melanoma and liver cancer metastasis in earlier years. For example, several key clock genes (BMAL1, CRY1 and CRY2) play a direct role in glioblastoma antitumor processes [209, 226]. There is a strong link between circadian rhythms and glioma genesis through the PI3K/AKT pathway [11]. This pathway, serving as a pivotal hub for pro-survival and proliferation signals within cells, exhibits abnormal activation as one of the hallmark events in the development and progression of gliomas (Fig. 5). Daily changes in glutathione levels have been previously reported, and glutathione is a regulator of REDOX status regulated by rhythmic expression of glutathione substance transporters [210]. There is growing evidence that circadian rhythms can influence glutathione secretion. For example, in a biological clock knockout mouse model, plasma glutathione levels fluctuated abnormally during the 24-h cycle, suggesting that circadian rhythms may influence glutathione secretion by regulating the expression of related genes [211, 212]. Glutathione-related enzymes, such as GCL, GR, glutathione peroxidase, and glutathione S-transferase, have been shown to be regulated by the circadian clock [212].

Gliomas are similarly involved in the alteration and disruption of circadian rhythms [213]. Clinically, it has been found that during the treatment of glioma patients, due to the vicious cycle of fatigue and lack of activity during the day, sleep is impaired at night, resulting in circadian rhythm disturbance. Moreover, experiments have confirmed that mice implanted with LN229 human glioma cells show an increase in endogenous cycle of the biological clock [214]. The neural network between circadian rhythms and gliomas is a complex area of research, and further studies are needed to gain a deeper understanding of the mechanisms involved.

## Technological and analytical advances

### Omics and time-series approaches to circadian research

In recent years, significant advancements in omics technologies, such as genomics, proteomics, metabolomics, and transcriptomics, have revolutionized the study of circadian rhythms [215, 227]. By enabling the high-throughput analysis of biological molecules, these approaches have provided new insights into the molecular mechanisms underlying circadian regulation. Time-series analyses, in particular, have become invaluable in understanding the dynamic nature of circadian processes. These approaches allow researchers to track the expression of clock genes and their downstream targets across the day-night cycle, shedding light on how circadian rhythms govern various biological functions. Time-series data, when combined with advanced computational models, can identify critical time-dependent changes in gene expression, protein synthesis, and metabolite levels, offering a more detailed view of circadian biology [228].

Recent studies have utilized omics techniques in conjunction with time-series approaches to explore circadian regulation at different levels of biological organization [229]. For example, in cancer research, integrating transcriptomics and time-series analysis has led to the identification of circadian-controlled genes that regulate cell cycle progression and tumor growth [230]. Similarly, in metabolic research, metabolomics paired with time-series data has revealed circadian oscillations in metabolites such as glucose, lipids, and amino acids, which are crucial for energy metabolism and disease pathogenesis [231, 232]. These studies highlight the potential of omics and time-series approaches to uncover the intricate relationships between circadian rhythms and various physiological processes, contributing to a better understanding of their role in health and disease.

Furthermore, as circadian biology is increasingly recognized for its therapeutic potential, omics and time-series data are being employed to improve personalized chronotherapy. By identifying circadian profiles of individual patients, researchers can tailor drug delivery and treatment timing to synchronize with the patient's biological rhythms, potentially enhancing therapeutic efficacy and reducing side effects [233]. This personalized approach is particularly relevant in cancer treatments, where optimizing the timing of chemotherapy based on circadian rhythms has shown promise in improving treatment outcomes and minimizing toxicity.

### Single-cell and spatial transcriptomic profiling of rhythmicity

Recent advancements in single-cell RNA sequencing and spatial transcriptomics have greatly expanded our understanding of circadian rhythms, allowing for more precise analysis of gene expression across diverse cell types in both temporal and spatial dimensions [234, 235]. Single-cell RNA-seq enables the identification of rhythmic gene expression profiles at the individual cell level, shedding light on how circadian oscillations are maintained within heterogeneous tissues and organs [236]. By studying the temporal changes in gene expression, researchers can determine how specific cell types, including immune cells, tumor cells, and neurons, synchronize their biological clocks to coordinate tissue-specific functions. This technique has revealed how circadian clock genes like BMAL1, CLOCK, and PER2 regulate cellular activities, such as cell division, metabolism, and apoptosis, in a time-dependent manner. Moreover, single-cell RNA sequencing has highlighted the differential circadian regulation in cancer cells, providing new insights into tumor growth, immune evasion, and metastasis.

### Chronobiology-informed computational and systems modeling

Recent advances in computational modeling and systems biology have significantly enhanced our understanding of circadian rhythms, allowing for a seamless integration of chronobiology with computational tools to analyze complex biological systems [229]. These systems models, which incorporate high-throughput omics data and time-series analysis, provide a powerful framework to simulate how circadian clocks influence various physiological processes, such as cell division, metabolism, and immune response [237]. By combining these advanced modeling techniques with extensive biological data, researchers can better understand how circadian rhythms interact with disease mechanisms like cancer proliferation, immune dysfunction, and metabolic disorders.

For instance, in cancer research, computational models have been instrumental in exploring how circadian rhythms regulate tumor growth and influence the effectiveness of cancer treatments [238]. These models have revealed that tumor cells exhibit circadian fluctuations in gene expression, which can affect their response to chemotherapy. By using chronobiology-informed computational approaches, we can now develop more personalized chronotherapies that align treatment timing with an individual’s circadian profile, ultimately optimizing therapeutic efficacy and reducing side effects. This shift toward personalized treatment, guided by circadian rhythm insights, represents a promising step forward in cancer care and other disease management.

### Emerging tools for real-time circadian monitoring in humans

The advent of wearable technologies and continuous monitoring tools has opened new possibilities for real-time circadian rhythm tracking in humans. These tools, such as wearable biosensors and smartwatches, enable the dynamic assessment of physiological parameters associated with circadian rhythms, including body temperature, heart rate, and sleep–wake cycles [239]. By continuously collecting data on these parameters, these devices provide valuable insights into an individual's circadian profile, which can be used to optimize health and wellness interventions. For example, recent advancements in wearable sensors have made it possible to track real-time changes in body temperature, which is a key circadian marker, and use this data to predict optimal times for physical activity, sleep, and medication administration [240].

Furthermore, emerging technologies now allow for the integration of multiple data streams, including light exposure, activity levels, and hormonal fluctuations (such as cortisol and melatonin), which are essential for understanding circadian disruptions. These systems combine real-time monitoring with machine learning algorithms to create personalized circadian profiles for users, enabling interventions that align with their biological rhythms. The ability to monitor and adjust circadian rhythms in real-time holds great promise for treating various conditions, including sleep disorders, metabolic diseases, and mood disorders, which are often exacerbated by circadian misalignment [241, 242]. For instance, circadian data gathered via wearables can be used to tailor chronotherapies that align with an individual’s internal clock, improving treatment efficacy and minimizing side effects [243].

## Translational and clinical perspectives

### Chronotherapy and personalized medicine

Cancer chronotherapy is a treatment method based on the principle of chronobiology, which involves the timing of pharmacologic and nonpharmacologic interventions, such as surgery, physical therapy, and psychotherapy [244]. Its core idea is to influence the occurrence, development and treatment effect of cancer by regulating the internal time rhythm of the organism [244]. In recent years, with the deepening of chronobiology research, cancer chronotherapy has made some progress in clinical application. Chronotherapy has been proven to be effective in improving drug efficacy and reducing drug toxicity [245]. Currently, clinical applications of temporal cancer therapies include the following:

Timed drug delivery: The circadian timing system (CTS) affects xenobiotics differently depending on the duration of exposure, and a number of recent clinical trials have demonstrated that timed regimens have a fivefold increase in drug tolerance and a doubling of drug efficacy compared to traditional non-timed regimens [246]. It is possible that radiation time therapy for cancer treatment in the morning reduces skin damage [247]. Thus, by timing drug administration, drugs can be used at the optimal time to improve cancer treatment efficacy and reduce side effects.

Treatment of biorhythmic disorders: In previous experiments we have shown that biorhythmic disorders are strongly associated with the development and progression of cancer. In experimental models, shift workers have a higher incidence of cancer, and breast cancer patients with sleep disorders tend to have shorter survival [248, 249]. Cancer can be prevented and treated by restoring normalcy to biological rhythms.

Synchronized chemotherapy: It has been found that tumor cells and normal cells have different time rhythms and that tumor cells are more active during certain time periods. Not long ago, a study showed that individuals with a low the circadian rhythm score (CRS) responded better to treatment and had a higher survival rate [250]. Therefore, by administering chemotherapy during the time period when tumor cells are most active, the effectiveness of chemotherapy can be improved and normal cell damage can be reduced.

The circadian clock can influence the metabolism of drugs and the temporal rhythm of drug action [251, 252]. The processes of drug metabolism and excretion in the body are regulated by the biological clock [253]. Therefore, the circadian clock can influence the concentration and timing of drug action in the body. The tolerability of multiple anticancer drugs can vary depending on the time of administration [254, 255]. Circadian rhythm has been shown to affect the changes of immune response, inflammatory response over time, and susceptibility to infection. Chronotherapy of cancer is to use the characteristics of the biological clock and circadian rhythm to select the best time for treatment, to achieve the purpose of cancer treatment effect and reduce side effects [256].

Numerous studies have demonstrated that temporal therapies improve the tolerance and antitumor efficacy of anticancer drugs in experimental animals as well as in cancer patients [257]. Circadian clock genes can affect the expression and activity of drug metabolizing enzymes. Many drug metabolizing enzymes are regulated by circadian clock genes and exhibit different expression levels and activities at different time points. Meanwhile, most of the key determinants of toxicity and efficacy of antineoplastic drugs have been shown to be regulated by circadian rhythms at the molecular level, and the core clock genes, BMAL1, and CLOCK, can be used to regulate drug metabolism through the regulation of drug metabolizing enzymes (e.g. CYPs and ABCG2) by regulating the transcriptional activity of drug metabolizing enzymes (e.g., CYPs and ABCG2) affecting drug toxicity [252, 258]. Overexpression of CRY1 has been reported to hinder the proliferation of glioblastoma stem cells (GSCs) in studies; therefore, pharmacological modulation of circadian components may be a novel strategy to improve tumor therapy [253]. In conclusion, a mutation or dysregulation of circadian clock genes may lead to changes in the expression and activity of drug-metabolizing enzymes, thereby affecting the metabolism and excretion of drugs in the body, and consequently drug efficacy and resistance.

Circadian clock genes can also affect the expression and activity of drug targets. Many drugs act on specific targets, and the expression and activity of the targets are also regulated by circadian clock genes. Circadian clock proteins control the transcription of drug metabolizing enzymes [258]. For example, BMAL1 regulates the expression of CYP3A11, which is involved in xenobiotic/drug metabolism, during the day. If CYP3A11 expression ceases, the toxicity of certain drugs is exacerbated [259, 260]. The circadian clock regulator ARNTL has been shown to be functionally involved in the resistance of high-risk primary prostate cancer (PCa) to ENZ, one of the most commonly used AR-targeted drugs, by driving the proliferation of tumor cells [260]. Temozolomide (TMZ) is a standard chemotherapy drug used to treat glioblastoma, and the maximum efficacy of TMZ occurs during a six-hour treatment window, corresponding to peaks in the core clock protein BMAL116, which occur before dawn in some human tissues. Therefore, taking TMZ in the morning is more in line with the circadian rhythm and the efficacy is better [261]. Since the cytotoxicity of anticancer drugs depends on their metabolism and detoxification, as well as on cell cycle-related targets, apoptosis, and DNA repair, which are controlled by circadian rhythms, the optimal time of administration can be chosen to maximize the efficacy of anticancer drugs [262].

Normally, neurons maintain the stability of the nervous system and the body's internal environment by regulating the release of neurotransmitters and neurohormones. However, during the development of cancer, abnormalities can occur in the nervous system, leading to disturbances in neuronal activity. Tumors typically secrete cytokines such as interleukin-6 (IL-6), which may regulate the circadian metabolism of distant organs. For example, IL-6 affects the insulin signaling pathway in the liver of mice carrying lung tumors [263]. Breast cancer can cause abnormal activity of calcitonin/orexin neurons through the sympathetic nerve of mice, thus leading to the destruction of sleep wake cycle and changes in glucose metabolism [264, 265]. It is particularly important that the sleep awakening cycle can be independent of the circadian rhythm. Therefore, even during the light dark cycle, sleep deprivation disrupts the daily rhythm of synaptic protein expression in the brain. Sleep fragmentation seems to be associated with tumor development, with day and night disruption and sleep disorders at least partially independently and synergistically promoting tumor development.

Anticancer chemotherapy may also help disrupt the host's circadian metabolism. Exogenous, long-acting glucocorticoids are usually used as part of chemotherapy regimens, which can cause daily glucose homeostasis disorders [266]. Psychological stress related to chemotherapy and adverse reactions such as nausea and vomiting may affect sleep and the host's circadian rhythm [267]. These observations indicate that tumors transmit profound signals to the physiology and metabolism of host organisms. Overall, it can be inferred that the disruption of the circadian rhythm of host metabolism caused by tumors or treatment further supports the progression of cancer, thereby creating a vicious cycle between the tumor's autonomic clock and the circadian rhythm of host metabolism. This disturbed neuronal activity may affect the sensitivity of tumor and normal cells to the drug, which in turn affects the effectiveness of temporal therapies. Circadian rhythms also play a key role in drug pharmacodynamics, with experiments confirming that the lethal toxic dose of a therapeutic drug varies significantly throughout the day.

We mentioned in the last section that glioblastoma (GBM) is the most common primary malignant brain tumor in adults, and temozolomide (TMZ) is one of the main drugs used in clinical trials for the treatment of GBM, due to the fact that GBM is highly resistant to chemotherapy is one of the reasons for the low survival rate of patients [268]. It was found that neuronal precursor cells express developmental downregulation 4–1 (NEDD4-1), which is significantly upregulated in GBM, and indole-3-methanol (IC3) treatment reversed chemoresistance in TMZ-resistant cells by inhibiting NEDD4-1-induced degradation of PTEN, which ultimately inhibited the AKT/NRF2/HO-1 signaling pathway. Transcriptional activation of endogenous NEDD4-1 degraded PTEN expression, enabling the NRF2/HO-1 antioxidant signaling response, which in turn made GBM cells resistant to temozolomide (TMZ) chemotherapy [269].

The cytotoxicity of anticancer drugs is determined by their metabolism and detoxification, as well as by their cell-cycle-related targets, apoptosis, and DNA repair, which are controlled by the biological clock. BMAL1 expression is up-regulated in patients with high-grade gliomas, and the expression of REV-ERB and CRY agonists, melatonin, or curcumin-assisted temozolomide (TME) treatment of gliomas reduces proliferation, migration, and invasion of U87MG glioblastoma cells through upregulation of BMAL1 expression and consequently downregulation of the p-AKT and METALLOPROTEINASE-9 pathways. The circadian rhythm and the occurrence of emotions. There may be a common genetic foundation [4]. The secretion of melatonin has obvious circadian rhythm. Melatonin as a circadian rhythm. The signal and sleep markers, when the patient's circadian rhythm is disrupted, it can be triggered by regulating the biological clock or affecting the expression of neurotrophic factors emotional issues such as depression, anxiety, and psychological pain [23, 26]. Compared to cortisol changes in circadian rhythms related to lactation have also been proven to be associated with metastatic or late lactation. The psychological status of patients with adenocarcinoma is related [27]. Horne et al. found that the sleep duration is related to the functional connectivity strength of emotion related brain regions [270, 271], and furthermore, it is pointed out that nighttime individuals may experience strong activation of the amygdala due to excessive activation. Inhibiting the activity of the cerebral cortex involved in emotion processing and triggering emotional disorders higher rate. Li Tingting et al. found that changes in sleep rhythm can affects the maturation of normal neural circuits, while synaptic pruning and improper refinement is related to the occurrence of psychological problems such as depression [172]. The circadian rhythm may indirectly affect the psychological recovery of cancer patients through factors such as cancer-related symptom clusters, sleep patterns, emotional regulation, and depression, as well as personality traits. Cancer correlation symptom cluster refers to the collection of symptoms such as pain, depression, fatigue, and sleep disorders during the diagnosis and treatment of cancer.

### Lifestyle and behavioral interventions

Recent advances in circadian biology have highlighted the importance of lifestyle and behavioral interventions in regulating circadian rhythms to improve health outcomes. Behavioral strategies such as optimizing sleep–wake cycles, exercise routines, and light exposure have been shown to help synchronize the biological clock, thus reducing the risk of chronic diseases associated with circadian disruption [272, 273]. For instance, proper light exposure during the day and reduced exposure to artificial light at night can help regulate the sleep–wake cycle, improving sleep quality and metabolic functions. Such lifestyle changes have been linked to improvements in conditions like obesity, diabetes, and cardiovascular diseases [274, 275]. Evidence suggests that regular exercise, when performed at specific times in alignment with the circadian rhythm, can enhance its benefits, including improved insulin sensitivity and reduced inflammatory responses [276, 277].

In addition to physical interventions, behavioral therapies such as cognitive-behavioral therapy (CBT) for insomnia and chronotherapy have shown promising results in improving circadian health [278]. Chronotherapy, which involves the timing of drug administration to align with the circadian rhythm, has been successfully applied in cancer treatment. Research has demonstrated that the timing of chemotherapy can significantly affect the efficacy of treatment, with better outcomes observed when drugs are administered at times that correspond to the tumor’s circadian vulnerability [279]. By adopting personalized chronotherapies that consider individual circadian rhythms, clinicians can enhance treatment outcomes, minimize side effects, and improve overall patient quality of life.

### Pharmacological modulation of clock components

The pharmacological regulation of circadian rhythm components has become a promising strategy for treating various diseases, especially cancer. The circadian rhythms regulates many biological processes, including cell cycle progress, metabolism and immune responses. The disruption of these rhythms can affect the progression of diseases and the therapeutic effect [280]. Recent studies have shown that drugs targeting core biological clock proteins such as BMAL1, CLOCK, PER and CRY can potentially influence cancer treatment outcomes by enhancing drug efficacy and reducing side effects [281, 282]. For instance, altering the expression or activity of the core clock gene BMAL1 has been proven to regulate the sensitivity of tumor cells to chemotherapy, thereby providing a new approach to enhance therapeutic outcomes [283]. By using drugs that affect the expression or stability of biological clock genes, this regulation may help optimize cancer treatment and synchronize treatment management with the patient's circadian rhythm cycle, also known as biological clock therapy.

Moreover, drugs that target circadian regulators have been explored for their potential in managing disorders like diabetes, obesity, and neurodegenerative diseases [284, 285]. These pharmacological interventions are being studied for their ability to reset the biological clock, synchronize physiological processes, and ultimately improve health outcomes. The success of such interventions underscores the growing importance of incorporating circadian biology into clinical practice, paving the way for personalized chronotherapy.

### Clinical trial design integrating circadian principles

Chronotherapy shows clear promise in optimizing therapeutic efficacy and reducing toxicity, but its translation into clinical practice remains constrained by challenges posed by significant interindividual heterogeneity in circadian rhythms. This heterogeneity stems from multi-level interacting factors: intrinsic factors include age, gender, genetic polymorphisms (such as variations in core clock genes like *CLOCK* and *BMAL1*), and baseline states of the neuroendocrine system; extrinsic factors involve lifestyle, such as shift work, nighttime light exposure, meal timing, and comorbid conditions, such as metabolic syndrome, sleep disorders [286, 287]. These factors collectively shape highly personalized profiles of patients' circadian systems in terms of phase, amplitude, and stability, directly determining variations in metabolism, tolerance, and response to time-dependent drugs. Conversely, tumor tissues are not passive targets of regulation; they may harbor disrupted or remodeled molecular clocks, whose rhythmic expression patterns, such as the rhythmic expression of genes like PER2 and CRY1, often evolves dynamically with tumor type, molecular subtype, pathological grade, and immune microenvironment characteristics [287]. This transforms the definition of the “optimal treatment window” from a single point in time into a complex function requiring the integration of both host and tumor circadian clock states [68, 288]. To address this challenge, future research must urgently evolve toward personalized, dynamic timing strategies. This requires integrating multidimensional continuous monitoring data: capturing behavioral rhythms (e.g., activity-rest cycles, body temperature fluctuations) via wearable devices; quantifying bodily fluid biomarkers, such as circadian secretion profiles of cortisol and melatonin, through periodic biological sampling; and attempting to indirectly assess tumor tissue clock characteristics via liquid biopsy or imaging techniques. Building upon this foundation, constructing personalized “host-tumor clock profiles” will become feasible. Furthermore, integrating systems biology models with machine learning algorithms to dynamically simulate and predict the time-pharmacokinetic/pharmacodynamic relationships of drugs holds promise for tailoring truly “personalized time prescriptions” to individual patients and their specific tumor characteristics. This strategy not only maximizes therapeutic indices within specific time windows but also provides theoretical foundations for temporal coordination among components in combination therapies, such as chemotherapy-immunotherapy-targeted therapy). It will drive the evolution of chronotherapy from universal principles toward precision medicine paradigms, ultimately enhancing overall treatment efficacy and patient quality of life.

### Drug resistance and recurrence

Treatment resistance is a great challenge in cancer clinical management, and the circadian rhythm plays an important regulatory role in this process. Drug resistance has become a growing problem in medicine today. Many traditional drugs for certain diseases often could not achieve the expected effect, and sometimes even completely ineffective. This phenomenon is not limited to bacteria and viruses, but also includes cancer and other complex diseases. Circadian rhythms regulate key biological processes that affect metabolic pathways. A large body of evidence suggests that the biological clock can modulate the effects of anticancer treatments, clock genes may be closely related to the mechanism of tumor drug resistance. For example, it has been reported that overexpression of BMAL1 increased the sensitivity of paclitaxel in squamous carcinoma of the tongue, and oxaliplatin and bevacizumab in colorectal cancer [289, 290]. However, silencing BMAL1 can also increase the sensitivity of breast cancer cells to cisplatin and doxorubicin [290]. Studies have shown that the circadian regulatory factor ARNTL is functionally involved in resistance to ENZ, one of the most commonly used targeted drugs, in high-risk primary prostate cancer by driving tumor cell proliferation [257]. Trastuzumab is the only her2-targeted drug used as standard first-line treatment for advanced her2-positive gastric cancer, but most patients inevitably develop resistance to trastuzumab, which has been experimentally demonstrated to lead to increased circadian rhythm expression of glycolysis-related genes compared with trastuzumab-sensitive cells. The clock genes BMAL1, CLOCK and PER1 can induce HK2 expression and circadian rhythm. Overall, the BMAL1-CLOCK-PER1 axis triggered trastuzumab resistance in TR cells by upregulating hk2-dependent glycolysis. Meanwhile, PER1 binds to PPARγ, which acts as a transcriptional activator, and is also able to regulate HK2 expression and rhythms, blocking trastuzumab resistance that can be reversed by PER1 expression in vivo [252]. In addition, the molecular clock interacts with the cell cycle and affects chemosensitivity and tolerance of 5-fluorouracil and oxaliplatin [254]. This implies that current antitumor therapeutic strategies should be considered in the biological clock dimension. Trastuzumab is a HER2-targeted drug for advanced HER2-positive gastric cancer, but has a high primary resistance rate. It has been found that the BMAL1-CLOCK-PER1 axis induced trastuzumab resistance in trastuzumab resistant gastric cancer cells by upregulating hexokinase 2-dependent glycolysis [252]. Therefore, pharmacological modulation of circadian components may be a new strategy to improve tumor therapy. The established link between circadian integrity and therapeutic response underscores the translational potential of circadian rhythms as prognostic and predictive biomarkers. Realizing this potential requires moving beyond observational correlations to developing actionable clinical tools. A pivotal step is the systematic definition of tumor-specific circadian disruption signatures, building upon the observation that the degree of clock gene misexpression correlates with cancer stage and aggression. This entails comprehensive profiling of rhythmic transcripts (e.g., PER2, BMAL1, CRY1) or circulating factors (e.g., melatonin, cortisol) across large, clinically annotated cohorts to identify robust patterns associated with disease progression and drug sensitivity. The validation of such signatures could lead to the development of integrated metrics, such as a Circadian Disruption Index (CDI), which quantifies the severity of circadian dysfunction in a given tumor. Prospective clinical studies are needed to determine whether a high CDI predicts poorer prognosis, higher risk of recurrence, or differential response to chronomodulated chemotherapy. Furthermore, non-invasive methods of circadian monitoring, such as tracking rest-activity rhythms via wearable devices, offer a promising avenue for dynamically assessing a patient’s circadian health and its interplay with treatment tolerance and efficacy. Ultimately, the convergence of tumor molecular clocks and systemic circadian physiology paves the way for a new class of biomarkers. These tools aim not merely for diagnosis but for dynamic prognosis and therapeutic guidance, enabling the stratification of patients for personalized chronotherapy and true integration of circadian biology into precision oncology paradigms.

## Concluding remarks and perspectives

Circadian rhythms are crucial to regulating various physiological functions, such as metabolism, immune responses, neural activity, and hormonal control [291]. Significant strides have been made in understanding the molecular and cellular foundations of these rhythms, especially regarding core clock genes and their feedback mechanisms. The interaction of circadian systems with metabolic, immune, and neural pathways has illuminated how internal biological timing influences health and disease [292]. This deeper understanding holds great promise for clinical applications, particularly for conditions like cancer, neurodegenerative diseases, and metabolic disorders where circadian disruption plays a significant role. Incorporating circadian principles into medical practice could pave the way for more precise and effective personalized therapies, enhancing patient care and outcomes.

However, the path from theory to clinical application still presents several challenges. While the integration of omics technologies, time-series data, and single-cell profiling has provided powerful tools for studying circadian rhythms, translating these findings into clinical settings remains complex. Personalized chronotherapy and the pharmacological modulation of circadian components require more targeted approaches [18]. Although emerging technologies, such as wearable devices and real-time circadian monitoring tools, offer substantial potential, individual variability in circadian rhythms necessitates highly customized treatment plans. Overcoming issues related to sensor accuracy, integration, and data reliability is key to fully realizing the potential of these innovative tools.

Looking ahead, future research should focus on optimizing clinical trial designs that incorporate circadian principles to assess their impact across a range of diseases. There is an urgent need for more individualized strategies that take into account the differences in circadian rhythms between patients. The use of computational models, real-time monitoring, and targeted interventions—whether pharmacological or lifestyle-based—will be essential for developing integrated treatment approaches. As our understanding of circadian biology deepens, we are well-positioned to enhance therapeutic outcomes by applying chronobiology-informed strategies, ultimately advancing healthcare to be more personalized, effective, and sustainable.

## Data Availability

Not applicable.
